# Immunogenic cell death and tertiary lymphoid structures jointly define dysregulated antitumor immune states and therapeutic vulnerability in lung adenocarcinoma

**DOI:** 10.3389/fimmu.2026.1917959

**Published:** 2026-09-30

**Authors:** Yuntian Lu, Yi Sun, Yihan Meng, Qinhua Huang, Yaoyi Zhang, Xiaorong Zhou, Jun Liu, Mengmeng Sang

**Affiliations:** 1 Department of Medical Oncology, Affiliated Hospital of Nantong University, Medical School of Nantong University, Nantong, China; 2 Department of Immunology, School of Medicine, Nantong University, Nantong, China; 3 Institute of Statistics and Big Data, Renmin University of China, Beijing, China

**Keywords:** immunogenic cell death, lung adenocarcinoma, machine learning, multilevel gene regulatory networks, tertiary lymphoid structures

## Abstract

**Background:**

Immunogenic cell death (ICD) and tertiary lymphoid structures (TLSs) are biologically distinct yet interconnected components of antitumor immunity. ICD promotes danger-associated immune activation, whereas TLSs organize local adaptive immune responses. However, how these processes jointly affect immune heterogeneity, prognosis, and therapeutic responsiveness in lung adenocarcinoma (LUAD) remains unclear.

**Methods:**

To define the coordinated roles of TLS- and ICD-related programs in LUAD, we used a multi-cohort and multi-layer analytical framework. This framework included transcriptomic analysis, Mendelian randomization, consensus machine-learning prognostic modeling, immune landscape characterization, single-cell RNA sequencing, multiplex immunohistochemistry, immunotherapy response assessment, exploratory drug screening, structural analyses, and multilayer network topology analysis.

**Results:**

TLS- and ICD-associated genes were transcriptionally dysregulated in LUAD. By integrating 13 machine-learning algorithms, we developed 801 candidate prognostic models and identified optimal TLS- and ICD-related models that stratified patients effectively into high- and low-risk groups. These models showed strong performance in TCGA and remained robust across six external cohorts. Low-risk tumors had higher TLS+ICD scores than high-risk tumors. Immune landscape analysis indicated that low-risk tumors had elevated immune scores, enhanced antigen presentation, cytotoxic T/NK-cell signatures, checkpoint activation, and lower Tumor Immune Dysfunction and Exclusion (TIDE) scores, suggesting a more immunotherapy-responsive microenvironment. Consistently, higher TLS+ICD scores correlated with improved survival and greater immunotherapy responsiveness in independent cohorts. Mendelian randomization and survival analyses prioritized CD40, CD5, and GFI1 as candidate TLS–ICD immune-regulatory genes, with single-cell and mIHC analyses defining their major cellular origins, spatial protein phenotypes, and prognostic relevance. Drug prediction, molecular docking, and molecular dynamics simulations identified CAY10576, SCH772984, and SZ4TA2 as potential therapeutic agents for high-risk LUAD. Multilayer gene regulatory network reconstruction and GLMY homology analysis revealed progressive disruption of higher-order immune-regulatory organization from normal to tumor tissues and between low TLS-risk/low ICD-risk (LL-risk) and high TLS-risk/high ICD-risk (HH-risk) states.

**Conclusions:**

TLS- and ICD-related programs jointly delineate clinically significant immune states in LUAD through convergent transcriptomic, genetic, single-cell, spatial, pharmacological, and systems-level evidence, and are associated with immune microenvironment remodeling, immunotherapy responsiveness, prognosis, and therapeutic vulnerability. These findings establish a biomarker-guided, machine learning-supported framework for LUAD risk stratification and may inform more precise immunotherapy-oriented therapeutic strategies.

## 1 Introduction

Lung adenocarcinoma (LUAD), a major histological subtype of non-small cell lung cancer (NSCLC), accounts for approximately 40% of all lung cancer cases and remains a leading cause of cancer-related death worldwide (1). Despite substantial advances in surgery, chemotherapy, targeted therapy, and immunotherapy, the prognosis of patients with advanced LUAD remains unsatisfactory, with a five-year survival rate of only around 4% (2–4). These persistently poor outcomes highlight the need for clinically relevant biomarkers and therapeutic targets that can improve risk stratification and support more precise treatment decisions.

Tertiary lymphoid structures (TLSs) are ectopic lymphoid aggregates that develop in non-lymphoid tissues under chronic inflammatory or persistent antigenic stimulation (5, 6). Structurally, TLSs resemble secondary lymphoid organs and are composed of organized immune cell populations, including B cells, T cells, dendritic cells, stromal cells, and follicular dendritic cells (7). Mature TLSs often contain segregated T-cell and B-cell zones and may develop germinal centers that support B-cell activation and differentiation (8). Functionally, TLSs serve as local immune niches for antigen presentation, lymphocyte priming, and adaptive immune amplification (6). In solid tumors, the presence of mature TLSs has been associated with enhanced antitumor immunity, increased infiltration of effector lymphocytes, improved clinical outcomes, and better responses to immune checkpoint blockade (9). Chemokine families such as CXC and CC chemokines are critically involved in TLS formation and immune-cell recruitment (10). In LUAD, accumulating evidence suggests that TLS abundance is associated with favorable prognosis and prolonged survival, supporting its potential relevance as both an immunological and translational biomarker (11, 12).

Immunogenic cell death (ICD) is a form of regulated cell death that stimulates adaptive immune responses against dying tumor cells (13). ICD is characterized by the release or surface exposure of damage-associated molecular patterns (DAMPs), including calreticulin (CALR) exposure, extracellular ATP release, and secretion of high mobility group box 1 (HMGB1) and type I interferons (14). These signals promote dendritic-cell maturation, antigen presentation, and T-cell activation, thereby enhancing tumor-specific immune responses (15). A range of anticancer treatments, including chemotherapy, radiotherapy, and photodynamic therapy, can induce ICD, and the combination of ICD-inducing therapies with immune checkpoint inhibitors may improve therapeutic efficacy and help overcome resistance (16). In LUAD, ICD-related pathways have emerged as potentially important mediators of tumor immune recognition, and ICD-associated genes such as CALR and HMGB1 may have prognostic and therapeutic relevance (17–19).

Multidimensional tumor heterogeneity, including spatial, temporal, and tumor microenvironmental heterogeneity, further shapes immune-cell organization, antigen presentation, immune evasion, and therapeutic response in LUAD (20). This broader framework supports the rationale for integrating TLS- and ICD-related signatures, as TLSs reflect spatially organized immune activation, whereas ICD captures cell-death-associated immune stimulation and antigen release. Although these two programs are closely linked to antitumor immunity, their coordinated roles within the heterogeneous LUAD tumor microenvironment remain insufficiently understood. We therefore hypothesized that these two programs jointly shape antitumor immune states in LUAD. In this study, we integrated multi-cohort transcriptomic analysis, consensus prognostic modeling, Mendelian randomization, single-cell RNA sequencing, multiplex immunohistochemistry (mIHC), exploratory drug screening, and systems-level network topology analysis to characterize the TLS–ICD axis in LUAD. By linking immune-risk stratification, cell-type-specific transcriptional programs, intercellular communication, spatial protein phenotypes, therapeutic vulnerability, and higher-order regulatory network organization, this study aimed to clarify how coordinated disruption of TLS- and ICD-related programs contributes to LUAD immune heterogeneity and disease progression.

## 2 Methods

### 2.1 Gene set and data collection

We curated 34 ICD-related genes (21–23) and 42 TLS-related genes (24, 25) from previously published literature. The complete gene sets are listed in **Supplementary Table 1**. RNA expression profiles and corresponding clinical metadata for normal and LUAD samples were obtained from The Cancer Genome Atlas (TCGA) (https://xenabrowser.net/), Genotype-Tissue Expression (GTEx) (https://www.gtexportal.org/home/), and Gene Expression Omnibus (GEO) (https://www.ncbi.nlm.nih.gov/geo/) databases. Seven datasets were included: TCGA-LUAD, GSE30219 (26), GSE31210 (27), GSE37745 (28), GSE50081 (29), GSE68465 (30), and GSE72094 (31). Immunohistochemistry (IHC) data of ICD- and TLS-related proteins were retrieved from the Human Protein Atlas (HPA) (https://www.proteinatlas.org) (32).

### 2.2 Differentially expressed gene identification and functional annotation

Differential expression analyses between LUAD and normal samples were conducted using the Voom method implemented in the *limma* package (33). Genes with an adjusted *p* < 0.05 and |logFC| > 0.5 were defined as DEGs. Volcano plots were used to visualize differentially expressed TLS- and ICD-related genes. Gene Ontology (GO) and Kyoto Encyclopedia of Genes and Genomes (KEGG) enrichment analyses were performed using *clusterProfiler* with default parameters (34). *P* values from enrichment analyses were adjusted for multiple testing using the Benjamini–Hochberg false discovery rate (FDR) method.

### 2.3 Cox regression analysis

Univariate Cox proportional hazards regression analyses were performed using the survival package (v3.2-7) to assess the prognostic value of TLS- and ICD-related genes in patients with LUAD from the TCGA cohort, with overall survival (OS) (35). Hazard ratios (HRs) and corresponding *P* values were calculated for each gene. For Kaplan–Meier (KM) survival analyses, patients were stratified into high- and low-expression groups according to the median expression level of each gene, and survival differences between groups were assessed using the log-rank test.

### 2.4 Construction of consensus prognostic models using machine learning

Thirteen machine learning algorithms were integrated to construct consensus prognostic models for LUAD survival prediction. These included artificial neural network (ANN) (36), survival random forest (Survival RF) (37), Lasso (38), Enet (39), supervised principal component (Supervised PCA) (40), extreme gradient boosting (XGBoost) (41), stepwise Cox regression (42), partial least squares regression cox (plsRcox) (43), gradient boosting decision tree (GBDT) (44), ridge regression (45), survival support vector machine (survival SVM) (46), Nu-support vector classification (Nu-SVC) (47), and light gradient boosting machine (LightGBM) (48).

For each model, a risk score was calculated for each patient according to the corresponding algorithm-derived coefficients or prediction framework. Patients were then classified into high- and low-risk groups using the median risk score within each cohort. Specifically, patients were dichotomized into high TLS-risk and low TLS-risk groups according to the median TLS-related risk score, and into high ICD-risk and low ICD-risk groups according to the median ICD-related risk score. Based on these two dichotomized risk scores, patients were further classified into four integrated TLS–ICD risk states: high TLS-risk/high ICD-risk (HH-risk), high TLS-risk/low ICD-risk (HL-risk), low TLS-risk/high ICD-risk (LH-risk), and low TLS-risk/low ICD-risk (LL-risk).

Model performance was assessed using time-dependent receiver operating characteristic (ROC) analysis, concordance index (C-index), and KM survival analysis in the TCGA discovery cohort and six external GEO validation cohorts. Final consensus models were selected primarily according to their C-index performance across the GEO validation cohorts, with priority given to models showing the most stable and reproducible predictive performance.

A prognostic nomogram was further constructed to estimate individualized 1-, 2-, and 3-year overall survival probabilities by integrating the TLS–ICD risk score with clinicopathological variables, including age, sex, and pathological stage. The nomogram was generated based on a Cox proportional hazards regression model using the rms R package (v6.4-0).

### 2.5 Identification of key prognostic genes

Gene importance was evaluated using five machine-learning algorithms, including support vector machine (SVM) (49), ANN, Boruta (50), random forest (RF) (51), and XGBoost. Standardized importance scores generated by each algorithm were averaged to derive an integrated importance score for each gene. Genes with consistently high integrated importance were prioritized as candidate prognostic genes for subsequent analyses.

### 2.6 Immune landscape analysis

The CIBERSORT algorithm (52), implemented in the IOBR package (v0.99.9) (53), was used to estimate the relative proportions of infiltrating immune cell populations from bulk transcriptomic data. The ESTIMATE package was applied to calculate ImmuneScore, StromalScore, and ESTIMATEScore for each patient. Gene set variation analysis (GSVA) (54) was further used to quantify tumor immune microenvironment (TIME)-related signatures (55, 56). Immune checkpoint expression data were obtained from Thorsson et al. (57). Potential response to immunotherapy was evaluated using the Tumor Immune Dysfunction and Exclusion (TIDE) database (58) and the Tracking Tumor Immunophenotype (TIP) database (59).

### 2.7 Single-cell RNA sequencing data analysis

Single-cell RNA-sequencing data from GSE131907 (60) were analyzed using the Seurat package (v4.4.0) (61). Low-quality cells were filtered according to the following criteria: nFeature_RNA > 200, nFeature_RNA < 5, 000, and percent.mt < 10%. After normalization using the NormalizeData function, highly variable genes were identified, and the data were scaled before principal component analysis (PCA). Batch effects were corrected using Harmony (v1.2.1) (62), and the Harmony-corrected embeddings were used for clustering and UMAP visualization. Major cell types were annotated according to canonical marker genes. Cells were assigned to TLS- and ICD-risk related subgroups (HH-risk, HL-risk, LH-risk, and LL-risk) according to risk scores derived from the optimal TLS and ICD prognostic models. The AddModuleScore function was used to quantify TLS- and ICD-associated expression signatures at the single-cell level. Differentially expressed genes were identified using the FindMarkers function with thresholds of *P* < 0.05 and |log2 fold change| > 0.25. Cell-cell communication networks were inferred using the CellChat package (v1.6.1) (63).

### 2.8 Mendelian randomization analysis

Potential causal associations between TLS-/ICD-related gene expression and LUAD risk were evaluated using the TwoSampleMR package (v0.6.18) (64). Expression quantitative trait locus (eQTL) data for TLS- and ICD-related genes were obtained from the IEU OpenGWAS database and used as exposure data (**Supplementary Table 2**). Four LUAD-related genome-wide association study (GWAS) datasets were included: ebi-a-GCST004744, ieu-a-984, ieu-a-965 (65), and finn-b-C3_NSCLC_ADENO (66). Independent single-nucleotide polymorphisms (SNPs) associated with exposure traits were selected as instrumental variables (IVs) using the thresholds *p* < 5×10^−4^ and *r²* < 0.001. Weak instruments were excluded using an F-statistic threshold > 10 (67). Potential confounding associations were screened using PhenoScanner (68).

Causal effects were primarily estimated using the inverse-variance weighted (IVW) method, with MR-Egger analysis used as a complementary approach. Effect estimates were reported as odds ratios (ORs) with corresponding 95% confidence intervals (CIs) and P values. For binary LUAD outcomes, MR estimates were converted to ORs, and 95% CIs were used to evaluate the precision of the estimated causal effects. Heterogeneity and horizontal pleiotropy were assessed using Cochran’s Q test and the MR-Egger intercept, respectively (69, 70). Associations with Q > 0.05 and pleiotropy *P* > 0.05 were considered to show no significant evidence of heterogeneity or directional pleiotropy.

### 2.9 Validation of CD5, CD40, and GFI1 expression and survival relevance

We first validated the single-cell expression patterns of CD5, CD40, and GFI1 across multiple LUAD single-cell RNA-sequencing datasets, including GSE99254 (71), GSE176021 (72), GSE149655 (73), GSE146100 (74), GSE139555 (75), GSE131907 (60), and GSE127465 (76).

To further validate tissue-level expression patterns, TSA-based sequential multiplex immunofluorescence/multiplex immunohistochemistry (mIF/mIHC) was performed on a lung adenocarcinoma tissue microarray (TMA; ZL-LUC1601; Shanghai Zhuoli Biotechnology Co., Ltd., China) (77). The following markers were simultaneously assessed: CD5, CD40, GFI1, CD3 (T-cell marker), CD45 (pan-immune marker), and pancytokeratin (PanCK; epithelial/tumor marker), with DAPI used as the nuclear counterstain. Anti-CD3 and anti-PanCK antibodies (BX50022 and BX50143, respectively) were purchased from Hangzhou Bailing (China). Anti-CD40 (40868T) and anti-CD5 (39300T) antibodies were obtained from Cell Signaling Technology (USA). Anti-CD45 (60287-1-Ig) was purchased from Proteintech (China), and anti-GFI1 (TA805544) was obtained from OriGene (USA).

Tissue sections were deparaffinized in xylene, rehydrated through graded ethanol, and subjected to antigen retrieval in alkaline AR9 buffer using a pressure cooker for 3 min. Endogenous peroxidase activity was blocked with 3% H_2_O_2_ for 15 min, followed by blocking with 5% bovine serum albumin for 30 min. Sequential staining was performed using a one-antibody-per-cycle protocol: CD40 (4 °C overnight; TSA-WH690), CD45 (37 °C for 1 h; TSA-WH620), GFI1 (4 °C overnight; TSA-WH570), CD3 (37 °C for 1 h; TSA-WH520), and CD5 (4 °C overnight; TSA-WH480). After each primary-antibody incubation, sections were incubated with an HRP-polymer secondary reagent for 50 min at room temperature, followed by TSA fluorophore deposition (1:300 dilution in TSA buffer for approximately 5–10 min). After each staining cycle, antibody complexes were stripped by heat-induced treatment in AR6 buffer at 95 °C for 25 min to minimize cross-reactivity and spectral overlap. PanCK was visualized using a 780-nm two-step detection system (RC780 A/B working solution for 10 min, followed by citrate buffer treatment at pH 6.0 and 95 °C for 20 min, and RC780C incubation for 15 min). Sections were then counterstained with DAPI for 15 min in the dark, mounted with antifade medium, and scanned at 20× magnification using a multispectral imaging system (77).

Image quantification was performed using APTime software (3DMedcare, v2.0.0) for TMA core segmentation, threshold setting, cell detection, and phenotype annotation. Protein-expression differences between paired normal and LUAD tissues were further quantified by digital image analysis (DIA) using the automated VIS-DIA VisioMorph platform (Visiopharm, Hørsholm, Denmark) (77). In addition to single-marker phenotypes (e.g., CD5+, CD40+, and GFI1+), composite phenotypes were defined according to single-cell co-expression and spatial co-localization, including CD40-CD5-related, CD40-GFI1-related, and CD5-GFI1-related phenotypes, as well as their contextual variants relative to CD3+, CD45+, or PanCK+ compartments.

Both absolute and relative quantitative metrics were analyzed. Absolute metrics were defined as the number or density of cells positive for a given phenotype within each tissue core, whereas relative metrics were calculated as ratios to CD3+, CD45+, or PanCK+ reference compartments. Differences between paired normal and LUAD tissues were assessed using paired two-group statistical tests, as appropriate. Associations between quantified tissue metrics and OS were further evaluated in LUAD patients. For survival analyses, patients were dichotomized into high- and low-level groups according to the optimal cutoff value for each quantified metric.

### 2.10 Drug prediction, molecular docking and molecular dynamics simulation

Drug-sensitivity data were retrieved from the Genomics of Drug Sensitivity in Cancer (GDSC) and Cancer Therapeutics Response Portal (CTRP) databases. The oncoPredict package (v0.2) was used to estimate the half-maximal inhibitory concentration (IC50) of candidate compounds in LUAD based on transcriptomic profiles (78). Potential therapeutic agents were prioritized according to the associations between predicted drug sensitivity and the expression levels of selected genes.

Protein structures of candidate target genes were obtained from the UniProt database (https://www.uniprot.org/) (79), and the chemical structures of predicted compounds were downloaded from PubChem database (https://pubchem.ncbi.nlm.nih.gov/) (80). Protein and ligand structures were preprocessed using UCSF Chimera. Molecular docking was then performed using DOCK (v6.10) to evaluate the binding modes and potential interactions between candidate compounds and target proteins (81).

Molecular dynamics simulations were performed in GROMACS (v2023) for 50 ns, with coordinates saved every 10 ps (82). The structural stability and dynamic behavior of the complexes were evaluated by calculating the root mean square deviation (RMSD), root mean square fluctuation (RMSF), radius of gyration (Rg), and solvent-accessible surface area (SASA). Simulation trajectories were visualized using GaussView and DuIvyTools (v0.4.0) (83).

### 2.11 Reconstruction of multilevel gene regulatory networks

Multilevel gene regulatory networks (GRNs) were reconstructed according to the quasi-dynamic game-graph theory (qdGGT) framework described by Sang et al. (84). TLS- and ICD-related genes were treated as components of a shared regulatory ecosystem with potential reciprocal interactions. Because the transcriptomic datasets used in this study were cross-sectional rather than longitudinal, ordinary differential equation (ODE)-based dynamic modeling could not be directly applied. We therefore used an allometric scaling strategy to transform discrete expression profiles into quasi-dynamic trajectories.

Let $x_{i\text{j}}\;$denote the expression level of gene *i* in sample *j*. For each sample *j*, the expression index ( $EI_{j}$) was defined as the total expression level across all analyzed genes as shown in Equation (1):

(1) $$EI_{j}=\sum_{i=1}^{p}G_{ij}$$

where *p* denotes the number of genes included in the network reconstruction. The expression pattern of each gene was then modeled as a function of *EI*, assuming an allometric part–whole relationship as shown in Equation (2):

(2) $$x_{i}(EI)=a_{i}EI^{b_{i}}+\epsilon_{i}$$

where $a_{i}\;$is the proportionality coefficient, $b_{i}$ is the allometric exponent, and $\epsilon_{i}\;$is the residual error term. This formulation allowed each gene to be represented by an *EI*-dependent quasi-dynamic expression trajectory.

To infer regulatory relationships among genes, a system of quasi-dynamic ordinary differential equations (qdODEs) was constructed. For a focal gene *i*, its expression change along the *EI*-dependent trajectory was modeled as the sum of an independent component and regulatory components contributed by other genes as shown in Equation (3):

(3) $$\frac{dx_{i}(EI)}{dEI}=f_{i}(EI;\theta_{i})+\sum_{k\neq \text{i}}\beta_{k\text{i}}g_{k}(EI;\theta_{k})+\epsilon_{i}(EI)$$

where $f_{i}(EI;\theta_{i})\;$represents the independent expression component of gene *i*, $g_{k}(EI;\theta_{k})\;$represents the regulatory contribution from gene *k*, and $\beta_{ki}\;$denotes the inferred regulatory effect of gene *k* on gene *i*. The independent component was modeled using nonparametric Legendre orthogonal polynomial functions, whereas the dependent components represented regulatory effects from other genes. The qdODE-derived coefficients were used to define the direction and strength of regulatory relationships between genes.

Because reconstruction of a fully connected GRN is computationally challenging and may introduce excessive noise, network sparsity and module-based reduction were applied. Sparse regulatory relationships were inferred using LASSO-based selection (38), and only non-zero qdODE-derived regulatory coefficients after sparsity selection were retained as directed regulatory edges. The magnitude of each retained coefficient was used as the corresponding edge weight.

To construct multilevel GRNs, genes were further grouped into modules according to the similarity of their *EI*-dependent quasi-dynamic expression trajectories (85). For each gene *i*, its paired expression trajectories across tumor and normal samples were jointly modeled using a multivariate functional clustering framework. The likelihood of the observed expression data was formulated as shown in Equation (4):

(4) $$L=\prod_{i=1}^{p}\sum_{m=1}^{K}\pi_{m}\phi (y_{i}|\mu_{m},{\text{Σ}}_{m})$$

where *K* denotes the number of clusters, $\pi_{m}\;$is the mixing proportion of cluster *m*, ϕ(·) denotes a multivariate longitudinal normal density function, $y_{i}\;$represents the paired expression trajectory of gene *i*, and $\mu_{m}\;$and ${\text{Σ}}_{m}\;$denote the cluster-specific mean trajectory and covariance structure, respectively. The mean trajectories were modeled using power functions, and the covariance structure was modeled using a bivariate first-order structured antedependence model, as previously described (86).

Model parameters were estimated using the expectation–maximization (EM) algorithm. The Bayesian information criterion (BIC) was used to determine the optimal number of modules. The posterior probability that gene i belonged to module m was calculated as shown in Equation (5):

(5) $$P(z_{i}=m|y_{i})=\frac{\pi_{m}\phi (y_{i}|\mu_{m},{\text{Σ}}_{m})}{\sum_{l=1}^{K}\pi_{l}\phi (y_{i}|\mu_{l},{\text{Σ}}_{l})}$$

Each gene was assigned to the module with the highest posterior probability. At the top layer, modules were treated as nodes to construct module-level regulatory networks. At the second layer, individual genes within selected modules were used as nodes to reconstruct gene-level regulatory subnetworks. This multilevel strategy allowed us to characterize both global module-level regulatory organization and local gene-level regulatory relationships across normal tissues, LUAD tissues, LL-risk samples, and HH-risk samples.

### 2.12 Topological analysis based on GLMY homology theory

To characterize the higher-order topological organization of the reconstructed GRNs, we applied the Grigor’yan–Lin–Muranov–Yau (GLMY) homology framework (87). GLMY homology extends classical homological analysis to directed graphs and enables the quantification of topological features in directed regulatory networks, including connected components, directed cycles, and higher-dimensional cavity-like structures.

For each biological group, including normal tissues, LUAD tissues, LL-risk samples, and HH-risk samples, a directed weighted graph was constructed as shown in Equation (6):

(6) G=(V,E,w)

where *V* denotes the set of nodes, *E* denotes the set of directed regulatory edges inferred from the corresponding GRN, and w:E→ℝ denotes the edge-weight function. Each edge weight was defined according to the qdODE-estimated regulatory coefficient, reflecting both the direction and magnitude of the inferred regulatory relationship.

To avoid reliance on a single arbitrary edge-weight cutoff, persistent GLMY homology was calculated across the full edge-weight filtration process. For each network, a filtration was constructed using the absolute values of the edge weights as shown in Equation (7). Let

(7) $$\lambda_{1}<\;\lambda_{2}<\;\cdots <\;\lambda_{M\;}$$

denote the ordered distinct absolute edge-weight values, where *M* is the total number of filtration levels $\lambda_{M\;\;}$. At filtration level, the filtered directed graph was defined as shown in Equations 8, 9:

(8) $$G_{\lambda_{M\;\;}}=(V,E_{\lambda_{M\;\;}})$$

Where

(9) $$E_{\lambda_{M\;\;}}=\{e\;\in E:|\text{w}(\text{e})\leq \lambda_{M\;\;}\}$$

As $\lambda_{M\;\;}$increased, edges were sequentially incorporated into the graph, generating a nested sequence of directed subgraphs. The resulting nested sequence of directed subgraphs is shown in Equation (10):

(10) $$G_{\lambda_{1}}\subseteq G_{\lambda_{2}}\subseteq \cdots \subseteq G_{\lambda_{M\;\;}}$$

Based on this filtration, persistent GLMY homology was computed to identify topological features that remained stable across multiple filtration levels. For two filtration levels $\lambda_{a\;\;}\leq \lambda_{b\;\;}$, the *k*-dimensional persistent GLMY homology group was defined as shown in Equation (11):

(11) $$H_{k}^{a,b}=Im(H_{k}(G_{\lambda_{a\;}})\to H_{k}(G_{\lambda_{b\;}}))$$

where $H_{k}(G_{\lambda_{M\;\;}})\;$denotes the *k*-dimensional GLMY homology group of $G_{\lambda_{a\;}}$, and the map is induced by the inclusion $G_{\lambda_{a\;}}\subseteq G_{\lambda_{b\;}}$. The corresponding persistent Betti number was calculated as shown in Equation (12):

(12) $$\beta_{k}^{a,b}=rank(H_{k}^{a,b})$$

In this study, we focused on $\beta_{0},$ $\beta_{1},\;$and $\beta_{2}$. Biologically, $\beta_{0}\;$reflects network connectivity or fragmentation, $\beta_{1}\;$represents directed cyclic or feedback-like regulatory structures, and $\beta_{2}\;$captures higher-order cavity-like organization beyond pairwise interactions. Persistent topological features were recorded according to their birth and death filtration levels, and longer persistence was interpreted as greater topological stability.

Persistent Betti curves were generated by tracking $\beta_{0},$ $\beta_{1},\;$and $\beta_{2}$ across filtration levels. Representative one-dimensional directed loops and two-dimensional higher-order structures with longer persistence were selected to highlight the major topological features embedded within the reconstructed GRNs. These features were interpreted as stable higher-order regulatory organizations rather than simply reflecting increased edge number or network density.

Because the analyzed networks contained a limited number of nodes after module-based reduction, the major low-dimensional GLMY features could be tracked and interpreted layer by layer. Under the weighted directed-graph and edge-sublevel filtration framework, persistent GLMY homology satisfies a stability property, such that small perturbations in edge weights induce bounded changes in persistence diagrams. The relevant mathematical definitions and stability theorem are provided in Reference (88).

### 2.13 Statistical analysis

The data analysis and plotting in this study were conducted via R software (version 4.1.0). Pearson’s correlation coefficient was used to assess the relationships between two continuous variables. Categorical variables were compared via the chi-square test, whereas the Wilcoxon rank-sum test or *t*-test was used to compare continuous variables.

## 3 Results

### 3.1 Expression patterns, functional annotation, and clinical relevance of TLS- and ICD-related genes

We first examined the differential expression of TLS- and ICD-related genes between normal lung tissues and LUAD samples. In total, 24 genes were upregulated and 20 genes were downregulated in LUAD relative to normal tissues (**Figure 1A**; **Supplementary Table 3**). Functional enrichment analysis showed that cytokine-mediated signaling pathways were the most significantly enriched biological processes among both upregulated and downregulated genes (**Figures 1B, C**; **Supplementary Tables 4**, **5**). KEGG analysis yielded similar results, with cytokine-cytokine receptor interaction emerging as the most significantly enriched pathway in both gene sets (**Figures 1D, E**; **Supplementary Tables 6**, **7**). These findings suggest that dysregulated TLS- and ICD-related genes are closely linked to cytokine-mediated immune signaling within the LUAD tumor microenvironment.

**Figure 1:**
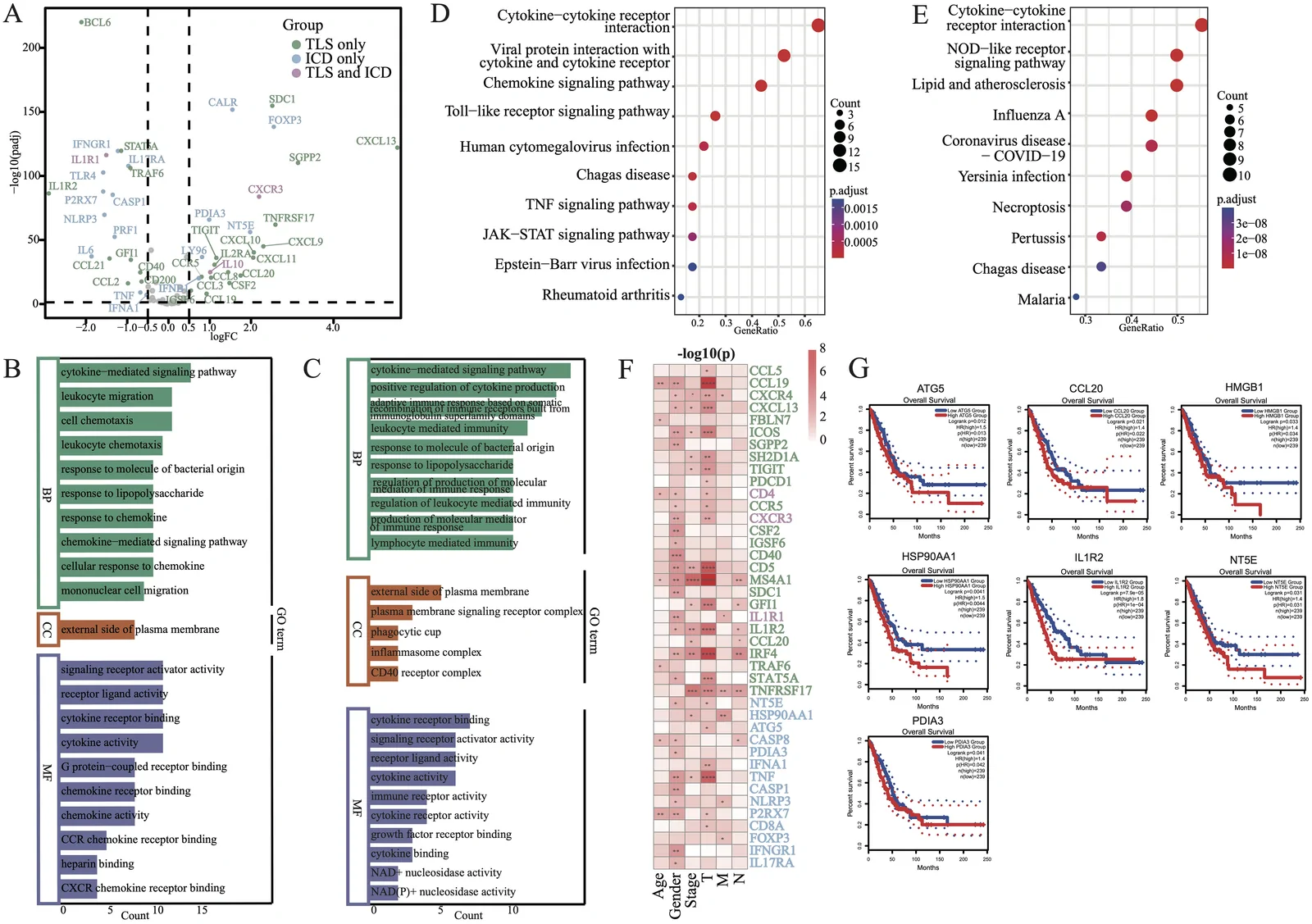
Differential expression, functional annotation, clinical relevance, and survival associations of TLS- and ICD-related genes in LUAD. **(A)** Differential expression of TLS- and ICD-related genes between normal lung tissues and LUAD samples. Gene Ontology (GO) enrichment analysis of **(B)** upregulated genes and **(C)** downregulated genes. Kyoto Encyclopedia of Genes and Genomes (KEGG) pathway enrichment analysis of **(D)** upregulated genes and **(E)** downregulated genes. **(F)** Differential expression of TLS- and ICD-related genes across clinical subgroups. **(G)** Kaplan–Meier curves showing the associations of representative prognostic TLS- and ICD-related genes with overall survival (OS) in patients with LUAD. *: P < 0.05, **: P < 0.01, and ***: P < 0.001.

We next assessed the clinical relevance of these genes across patient subgroups stratified by age, sex, stage, and TNM category. Seven genes differed significantly between younger and older patients (cutoff: 60 years; **Figure 1F**; **Supplementary Figure 1A**). Additional differences across sex, TNM stage, and overall pathological stage are shown in **Figure 1F** and **Supplementary Figures 1B–F**, among which *CXCR4*, *MS4A1*, *IL1R2*, *IRF4*, and *TNFRSF17* showed the most consistent variation across clinical subgroups.

Cox regression analysis further identified several prognostically relevant genes. Several genes were associated with worse OS, including ATG5, CCL20, HMGB1, HSP90AA1, IL1R2, NT5E, and PDIA3 (**Figure 1G**). In contrast, CD5, CD40, CXCR4, ENTPD1, GFI1, ICOS, IGSF6, IRF4, MS4A1, SH2D1A, TLR4, and TNFRSF17 were associated with favorable OS (**Supplementary Figure 2**). Together, these results indicate that TLS- and ICD-related genes are not only transcriptionally dysregulated in LUAD but also closely associated with clinical heterogeneity and patient prognosis.

### 3.2 Consensus prognostic modeling based on TLS- and ICD-related genes

To establish robust prognostic models for LUAD, we integrated 13 machine-learning algorithms and generated a total of 801 candidate models. TLS- and ICD-related genes were first modeled separately (**Supplementary Figures 3**, **4**), and the optimal models were then integrated to construct an integrated TLS-ICD framework. In the TCGA training cohort, model performance was evaluated using the C-index, and the mean C-index across six GEO validation cohorts was used to assess external robustness.

Among TLS-based models, the combination of survival random forest and XGBoost with a maximum depth of 5 showed the best overall performance (**Figure 2A**; **Supplementary Figure 3**). For ICD-based models, the optimal model combined survival random forest and supervised principal components analysis (**Figure 2B**; **Supplementary Figure 4**). Risk scores derived from the optimal TLS and ICD models stratified TCGA patients into high- and low-risk groups, with significantly poorer survival observed in the high-risk groups for both models (**Supplementary Figures 5A, C**).

**Figure 2:**
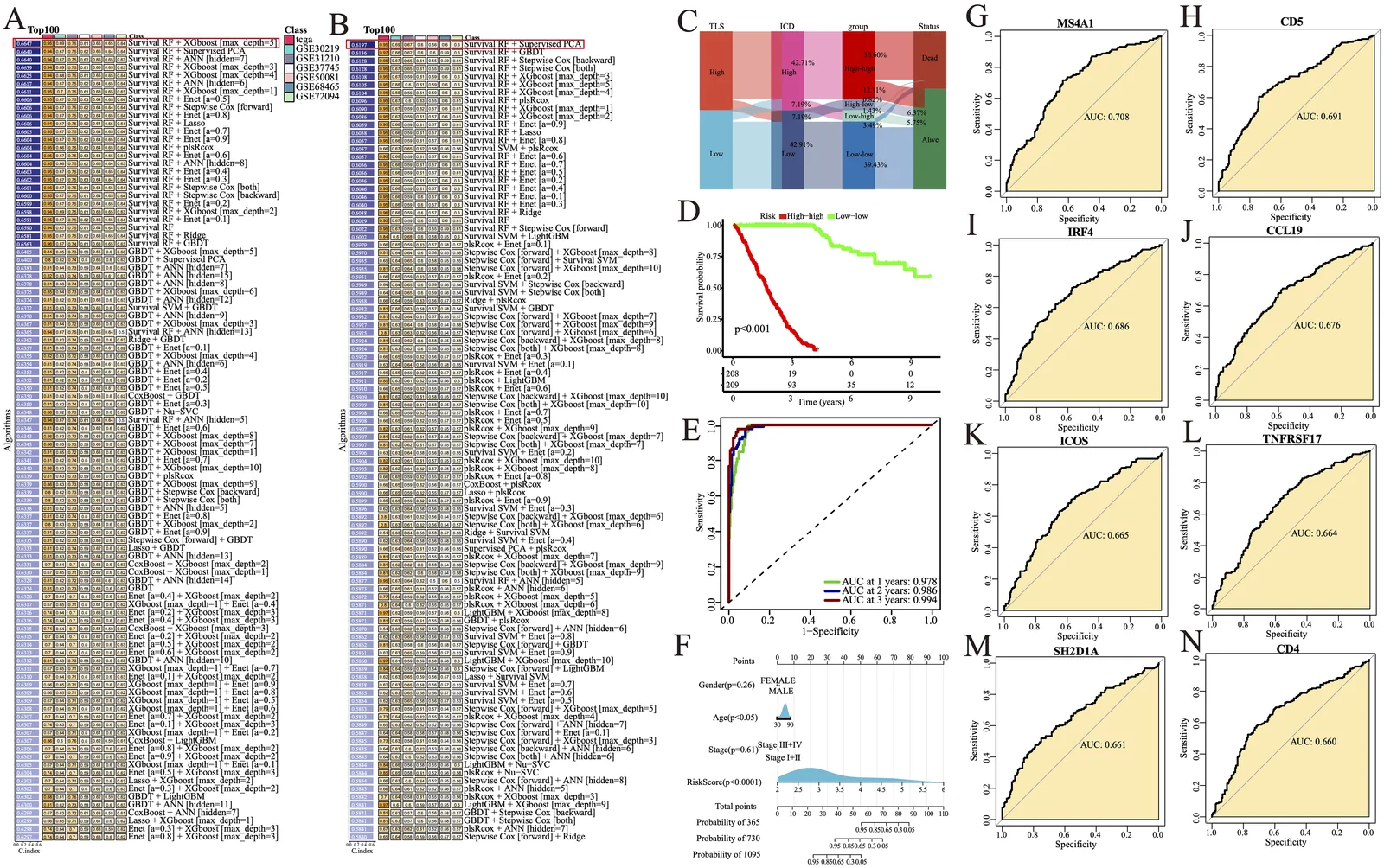
Construction and validation of TLS- and ICD-derived prognostic signatures using an integrative machine-learning framework. C-index values of the training and validation models for the top 100 prognostic signatures derived from **(A)** TLS-related genes and **(B)** ICD-related genes. **(C)** Patients were stratified into four subgroups according to TLS- and ICD-related risk scores: high TLS-risk/high ICD-risk (HH-risk), high TLS risk/low ICD risk (HL-risk), low TLS risk/high ICD risk (LH-risk), and low TLS risk/low ICD risk (LL-risk). The Sankey plot shows the proportions of alive and deceased patients across the four subgroups. **(D)** Kaplan–Meier survival curves comparing the HH-risk and LL-risk groups. **(E)** Time-dependent ROC curves showing the predictive performance of the integrated TLS–ICD model at 1, 2, and 3 years. **(F)** Nomogram integrating gender, age, pathological stage, and the integrated TLS–ICD risk score to predict 1-, 2-, and 3-year overall survival probabilities in LUAD patients. **(G–N)** ROC curves of the top eight genes with the highest discriminatory performance for distinguishing HH-risk from LL-risk tumors.

To further assess the joint prognostic impact of TLS and ICD programs, TCGA patients were categorized into four integrated TLS–ICD risk states: high TLS-risk/high ICD-risk (HH-risk), high TLS-risk/low ICD-risk (HL-risk), low TLS-risk/high ICD-risk (LH-risk), and low TLS-risk/low ICD-risk (LL-risk) (**Figure 2C**). Patients in the LL-risk group exhibited the most favorable survival, whereas those in the HH-risk group had the poorest outcomes (**Figures 2C, D**). Time-dependent ROC analysis showed excellent predictive performance. The TLS model achieved 1-, 2-, and 3-year AUCs of 0.976, 0.983, and 0.990, respectively (**Supplementary Figure 5B**), while the corresponding AUCs for the ICD model were 0.978, 0.986, and 0.991 (**Supplementary Figure 5D**). For the full four-state TLS–ICD stratification, the 1-, 2-, and 3-year AUCs were 0.982, 0.987, and 0.993, respectively (**Supplementary Figure 6A**). Because HH-risk and LL-risk represented the two most divergent risk states, we further performed an exploratory extreme-subgroup comparison, in which the 1-, 2-, and 3-year AUCs reached 0.978, 0.986, and 0.994, respectively (**Figure 2E**).

We next constructed a nomogram integrating the TLS–ICD risk score with clinicopathological variables, including age, sex, and pathological stage (**Figure 2F**). The TLS–ICD risk score contributed the largest proportion of total points and was significantly associated with survival probability. This nomogram provided an individualized visualization of 1-, 2-, and 3-year survival probability and further illustrated the prognostic contribution of the risk score in the full TCGA-LUAD population. We further identified the top eight genes that most effectively distinguished HH-risk and LL-risk tumors, namely *HSP90AA1*, *CCL19*, *IL1R2*, *MS4A1*, *IRF4*, *CD5*, *TNFRSF17*, and *IGSF6* (**Figures 2G–N**).

External validation in six GEO cohorts further supported the robustness of the modeling framework. For the individual models, significant survival differences between high- and low-risk groups were observed across all six datasets for the TLS model and in four datasets for the ICD model (**Supplementary Figure 7**). We then integrated the TLS and ICD models to define four risk states in each external cohort (**Figures 3A–F**). Kaplan–Meier analysis showed significant survival differences among the four risk states across all six external cohorts, with LL-risk patients generally showing the most favorable survival and HH-risk patients showing the poorest outcome (**Figures 3G–L**; **Supplementary Figures 6A–G**). For the full four-state stratification, the corresponding AUCs ranged from 0.559 to 0.805 (**Supplementary Figures 6H–N**). In the exploratory comparison between the two extreme risk states, HH-risk and LL-risk, the AUC values ranged from 0.69 to 0.862 across the external cohorts (**Figures 3M–R**). Together, these findings demonstrate that the integrated TLS–ICD framework provides reproducible prognostic stratification across independent LUAD cohorts, while the HH-risk versus LL-risk comparison highlights the prognostic contrast between the two most divergent immune-risk states.

**Figure 3:**
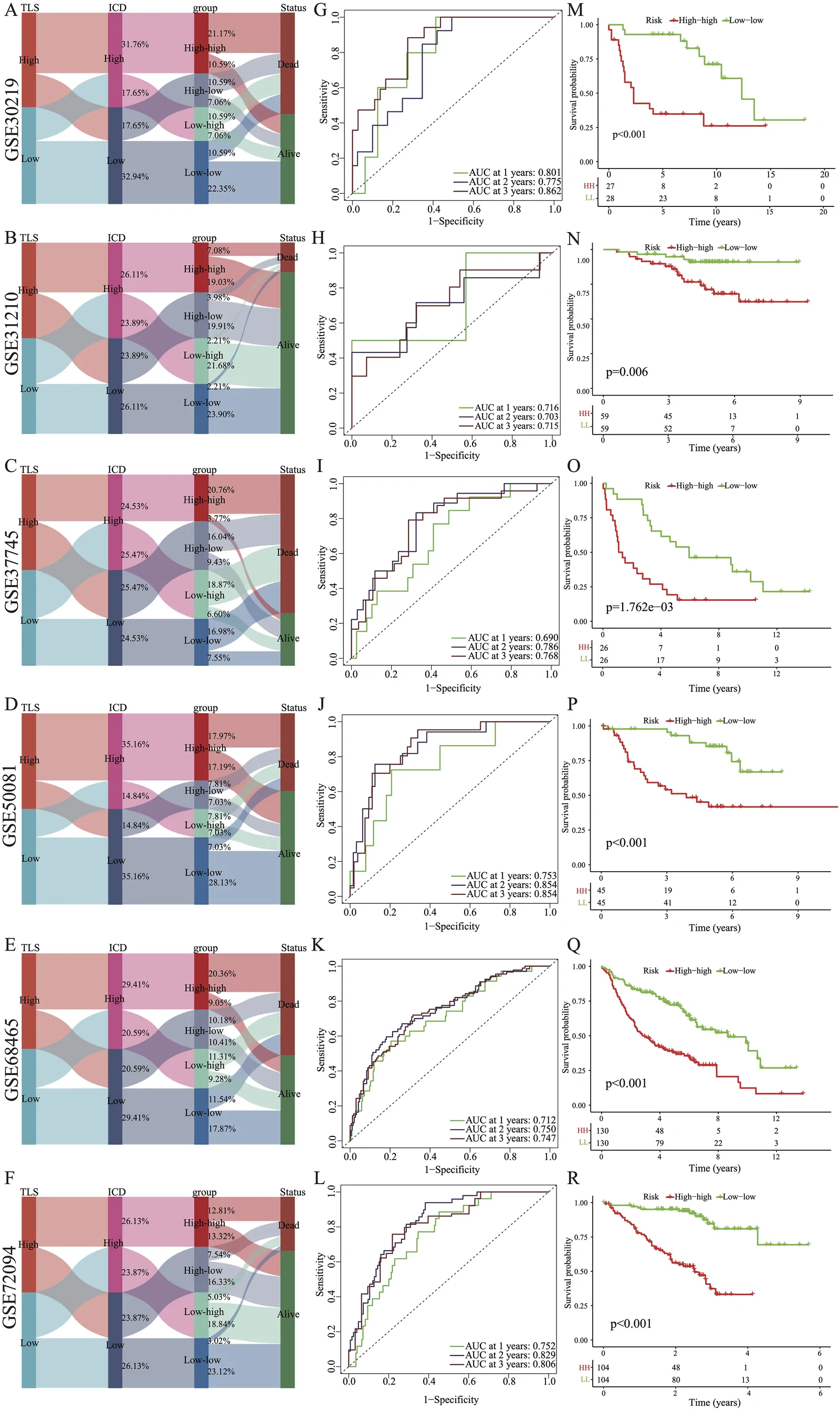
External validation of the integrated TLS–ICD prognostic framework in six GEO cohorts. Sankey plots showing the proportions of alive and deceased patients, Kaplan–Meier survival curves, and ROC curves in six independent GEO datasets: **(A, G, M)** GSE30219; **(B, H, N)** GSE31210; **(C, I, O)** GSE37745; **(D, J, P)** GSE50081; **(E, K, Q)** GSE68465; and **(F, L, R)** GSE72094.

To prioritize the most influential prognostic genes, five machine-learning methods, including SVM, ANN, Boruta, RF, and XGBoost, were applied (**Supplementary Figures 8A–F**). After normalization and integration of feature importance values, *CCL20*, *CCL21*, *IL1R2*, *MS4A1*, and *BAX* emerged as the top five genes most strongly associated with patient survival (**Supplementary Figure 8G**).

### 3.3 Immune landscape differences between HH-risk and LL-risk groups

We next compared the immune landscapes of the HH-risk and LL-risk groups. ImmuneScore, StromalScore, and ESTIMATE score were all significantly higher in the LL-risk group, indicating a more immune-enriched tumor microenvironment in low-risk tumors (**Figure 4A**). Immune-cell deconvolution further revealed distinct cellular compositions between the two groups. The LL-risk group showed higher infiltration of memory B cells, resting memory CD4 T cells, regulatory T cells, resting dendritic cells, and resting mast cells. By contrast, the HH-risk group showed increased proportions of activated memory CD4 T cells, M0 and M1 macrophages, activated mast cells, and neutrophils (**Figure 4B**).

**Figure 4:**
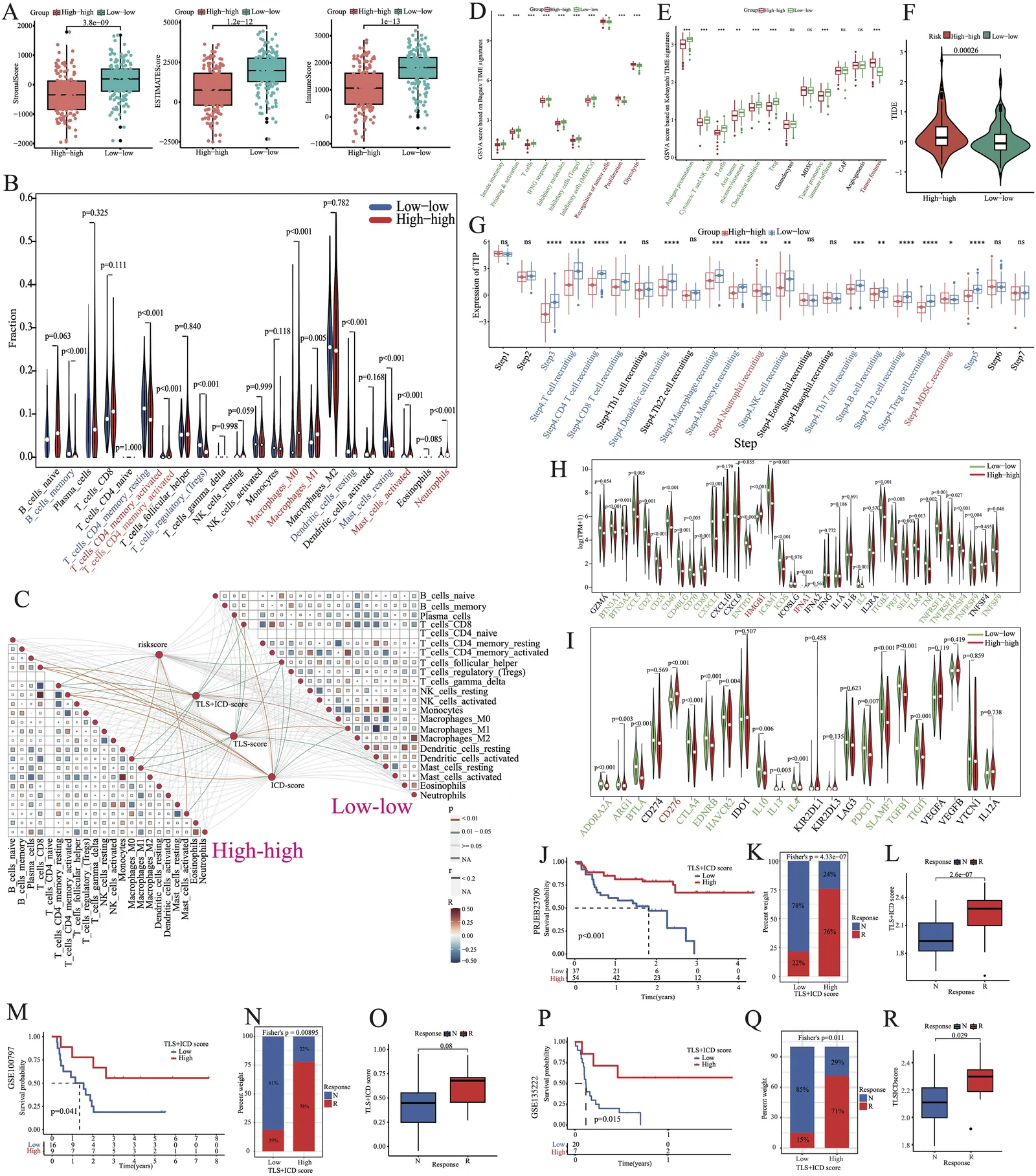
Immune landscape and immunotherapy relevance of the HH-risk and LL-risk subgroups in LUAD. **(A)** Comparison of ImmuneScore, StromalScore, and ESTIMATE score between the HH-risk and LL-risk groups. **(B)** Differences in infiltrating immune-cell populations between the HH-risk and LL-risk groups. **(C)** Correlation patterns among immune-cell populations in the two groups. GSVA-based comparison of tumor microenvironment (TME) signatures derived from the **(D)** Kobayashi and **(E)** Bagaev frameworks. **(F)** Comparison of TIDE scores between the HH-risk and LL-risk groups. **(G)** Differences in cancer-immunity-cycle activity between the two groups. Differential expression of **(H)** inhibitory and **(I)** stimulatory immune checkpoint genes. In three independent immunotherapy cohorts, PRJEB23709, GSE100797, and GSE135222, **(J, M, P)** Kaplan–Meier curves show the association between overall survival and the integrated TLS+ICD score, **(K, N, Q)** bar plots show the proportions of responders and non-responders in the high- and low-score groups, and **(L, O, R)** boxplots compare TLS+ICD scores between responders and non-responders. **: P < 0.01, ***: P < 0.001, and ****: P < 0.0001.

Cell-cell correlation patterns also differed substantially between the two risk states (**Figure 4C**; **Supplementary Tables 8**, **9**). In the HH-risk group, resting memory CD4+ T cells were negatively correlated with activated memory CD4+ T cells and CD8+ T cells, while M2 macrophages were positively correlated with monocytes and activated memory CD4+ T cells were positively correlated with CD8+ T cells. These associations were weaker in the LL-risk group. In contrast, dendritic cells were more strongly negatively correlated with M1 macrophages in LL-risk tumors, and the correlation between neutrophils and plasma cells showed opposite directions in HH-risk and LL-risk tumors. In addition, CD8+ T cells and activated memory CD4+ T cells were associated with TLS, ICD, and combined TLS+ICD scores in HH-risk tumors, whereas M1 macrophages were associated with all three scores in LL-risk tumors (**Supplementary Tables 10**, **11**).

To further characterize the tumor microenvironment, we evaluated previously defined TME-related signatures. The HH-risk group was enriched for tumor cell recognition, proliferation, glycolysis, and tumor-intrinsic programs. In contrast, the LL-risk group showed stronger innate immunity, priming and activation, T-cell signatures, IFN-γ response, antigen presentation, cytotoxic T/NK-cell programs, B-cell signatures, checkpoint inhibition, and an overall antitumor microenvironment (**Figures 4D, E**). These findings indicate that LL-risk tumors exhibit a more immune-active and potentially immunotherapy-responsive microenvironment than HH-risk tumors.

Consistent with this interpretation, the LL-risk group had significantly lower TIDE scores than the HH-risk group, suggesting a higher likelihood of response to immune checkpoint blockade (**Figure 4F**). Cancer-immunity cycle analysis further supported a more active immune state in LL-risk tumors across most steps, with the exception of neutrophil and MDSC recruitment, which was more pronounced in HH-risk tumors (**Figure 4G**). Moreover, most stimulatory and inhibitory immune checkpoint molecules were upregulated in the LL-risk group (**Figures 4H, I**). In three independent immunotherapy cohorts, PRJEB23709 (89), GSE100797 (90), and GSE135222 (91) patients with high TLS+ICD scores had better overall survival and a higher proportion of responders than those with low TLS+ICD scores (**Figures 4J–R**). Similar trends were observed for TLS or ICD scores alone (**Supplementary Figures 9A–R**). Together, these results support a cooperative role for TLS and ICD programs in shaping immunotherapy responsiveness.

### 3.4 Single-cell atlas of risk-state-specific immune programs and intercellular crosstalk in LUAD

To resolve TLS- and ICD-associated risk states at single-cell resolution, we analyzed scRNA-seq data from 11 early-stage LUAD samples in GSE131907, comprising a total of 45149 cells. Cells were classified into eight major cell types, including T lymphocytes, NK cells, myeloid cells, mast cells, fibroblasts, epithelial cells, endothelial cells, and B lymphocytes, on the basis of canonical markers (**Figures 5A, B**). Most ICD-related genes, including *CD4*, *IFNA1*, and *IFNB1*, were predominantly expressed in myeloid cells (**Figure 5C**), whereas many TLS-related genes, such as *CCR5*, *CXCL11*, and *CXCR3*, were mainly enriched in T lymphocytes (**Figure 5D**).

**Figure 5:**
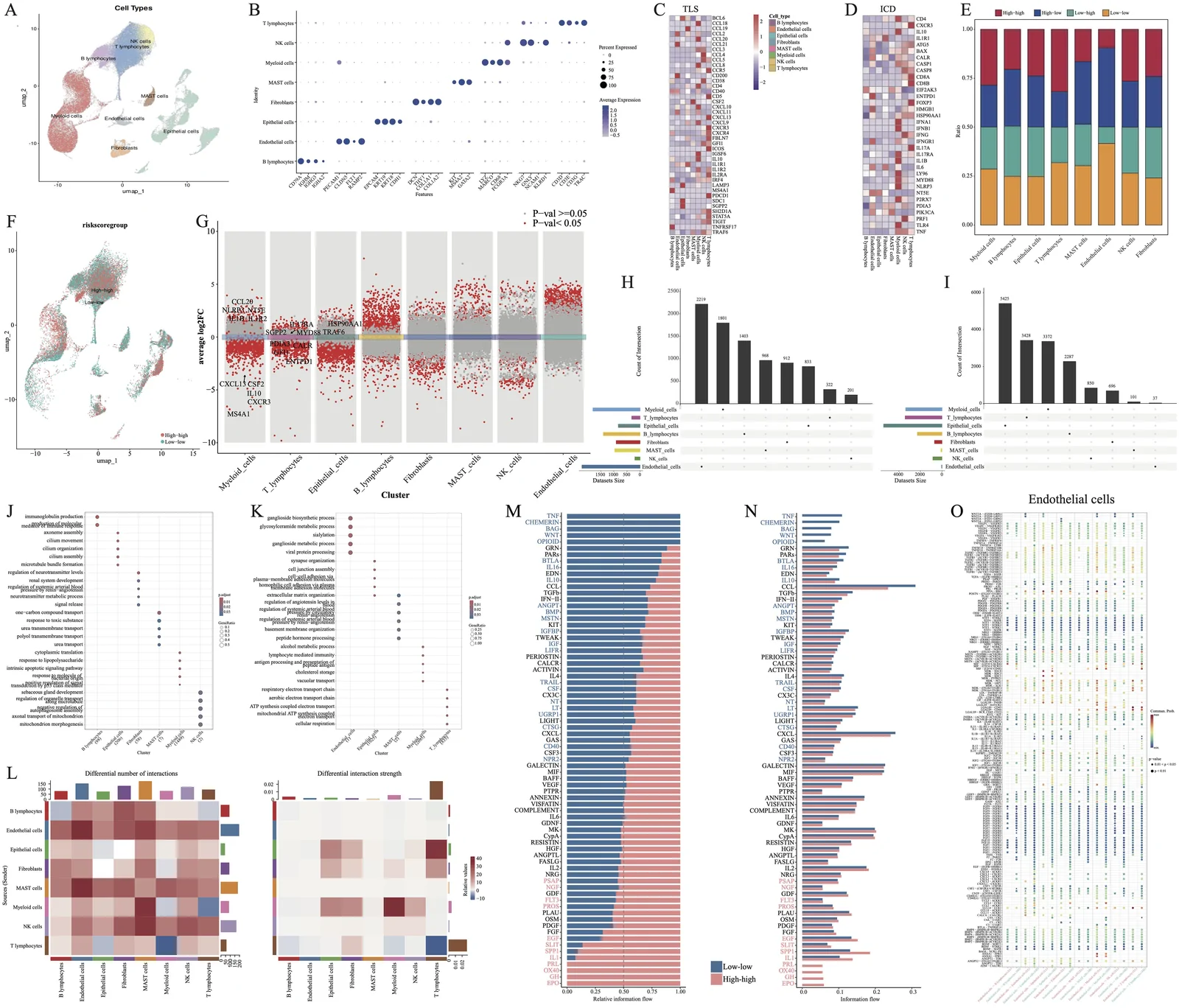
Single-cell characterization of TLS–ICD-associated risk states and intercellular communication in LUAD. **(A)** UMAP visualization of single cells from LUAD samples. **(B)** Annotation of major cell populations based on canonical marker genes. Expression patterns of **(C)** ICD-related genes and **(D)** TLS-related genes across cell types. **(E)** Proportions of each cell type in the HH-risk, HL-risk, LH-risk, and LL-risk subgroups. **(F)** UMAP visualization of cells assigned to the HH-risk and LL-risk states. **(G)** Differentially expressed genes (DEGs) between HH-risk and LL-risk cells in each cell type. UpSet plots showing the intersections of **(H)** upregulated and **(I)** downregulated DEGs across cell types. Gene Ontology enrichment analysis of **(J)** upregulated and **(K)** downregulated DEGs. **(L)** Differential number and strength of cell-cell interactions between the HH-risk and LL-risk groups. **(M)** Relative and **(N)** absolute information flow of signaling pathways in the two groups. **(O)** Comparison of selected ligand-receptor interactions between endothelial cells and other cell populations in the HH-risk and LL-risk groups.

To investigate how TLS- and ICD-related programs contribute to cellular risk states, we assigned each cell a TLS risk score and an ICD risk score using the optimal bulk transcriptomic prognostic models. Cells of each type were then stratified into HH-risk, HL-risk, LH-risk, and LL-risk groups according to the corresponding median risk scores (**Figures 5E, F**; **Supplementary Figures 10A–C**). This framework enabled us to compare transcriptional programs and intercellular communication patterns between the HH-risk and LL-risk cell states.

Differential expression analysis revealed marked cell-type-specific differences between HH-risk and LL-risk cells. For example, *CCL20*, *NLRP3*, and *NT5E* were upregulated in HH-risk myeloid cells, whereas *IL17RA*, *SGPP2*, and *MYD88* were enriched in HH-risk T lymphocytes. Differentially expressed genes across cell types are summarized in **Figures 5G–I** and **Supplementary Table 12**. Notably, TLS- and ICD-related genes were predominantly expressed in myeloid cells (**Supplementary Figures 10D, E**). Functional annotation further showed that upregulated and downregulated genes in HH-risk cells exhibited distinct, cell-type-specific biological programs (**Figures 5J, K**; **Supplementary Tables 13**, **14**). At the signature level, TLS scores were significantly increased in HH-risk endothelial cells but decreased in most other HH-risk cell types, whereas ICD scores were significantly lower in HH-risk T and NK cells but higher in other cell populations (**Supplementary Figures 10F, G**).

We next assessed intercellular communication using CellChat. Overall, the HH-risk group showed a greater number of inferred interactions but weaker interaction strength than the LL-risk group (**Supplementary Figures 10H–J**). At a finer level, endothelial cells in HH-risk tumors communicated more extensively with myeloid cells, NK cells, and mast cells, whereas interactions between myeloid cells and T lymphocytes were reduced (**Figure 5L**). Interaction strength between myeloid cells and between T lymphocytes and myeloid cells was increased in HH-risk tumors, while T-cell-to-T-cell communication was weaker (**Figure 5L**). Analysis of signaling pathway information flow showed that PRL, OX40, GH, and EPO signaling were specific to HH-risk tumors, whereas TNF, CHEMERIN, BAG, WNT, and OPIOID signaling were specific to LL-risk tumors (**Figure 5M**). Commonly active pathways in both groups included CCL, CXCL, GALECTIN, and MIF signaling (**Figure 5N**). At the ligand–receptor level, LL-risk endothelial cells exhibited extensive WNT3A–FZD8/LRP5 and WNT3A–FZD8/LRP6 signaling, which was absent in HH-risk tumors. By contrast, HH-risk endothelial cells preferentially showed HBEGF–ERBB4, HBEGF–EGFR, and HBEGF–(ERBB2+ERBB4) signaling (**Figure 5O**; **Supplementary Figure 11**). These data indicate that HH-risk and LL-risk tumors are characterized by distinct immune programs and cell-cell communication architectures.

### 3.5 Mendelian randomization analysis

To further assess causal relevance, we performed Mendelian randomization analyses for TLS- and ICD-related signatures and individual genes. The integrated TLS+ICD score, as well as the TLS score alone, showed protective associations with LUAD risk (**Figure 6**). At the gene level, *CD40*, *CD5*, *EIF2AK3*, *FBLN7*, *GFI1*, *LAMP3*, *P2RX7*, and *PDCD1* were identified as protective factors, whereas *IFNGR1*, *IL10*, and *IRF4* were associated with increased LUAD risk (**Figure 6**; **Supplementary Figure 12**). These results complement the transcriptomic and survival analyses by providing genetic support for the disease relevance of key TLS- and ICD-related genes.

**Figure 6:**
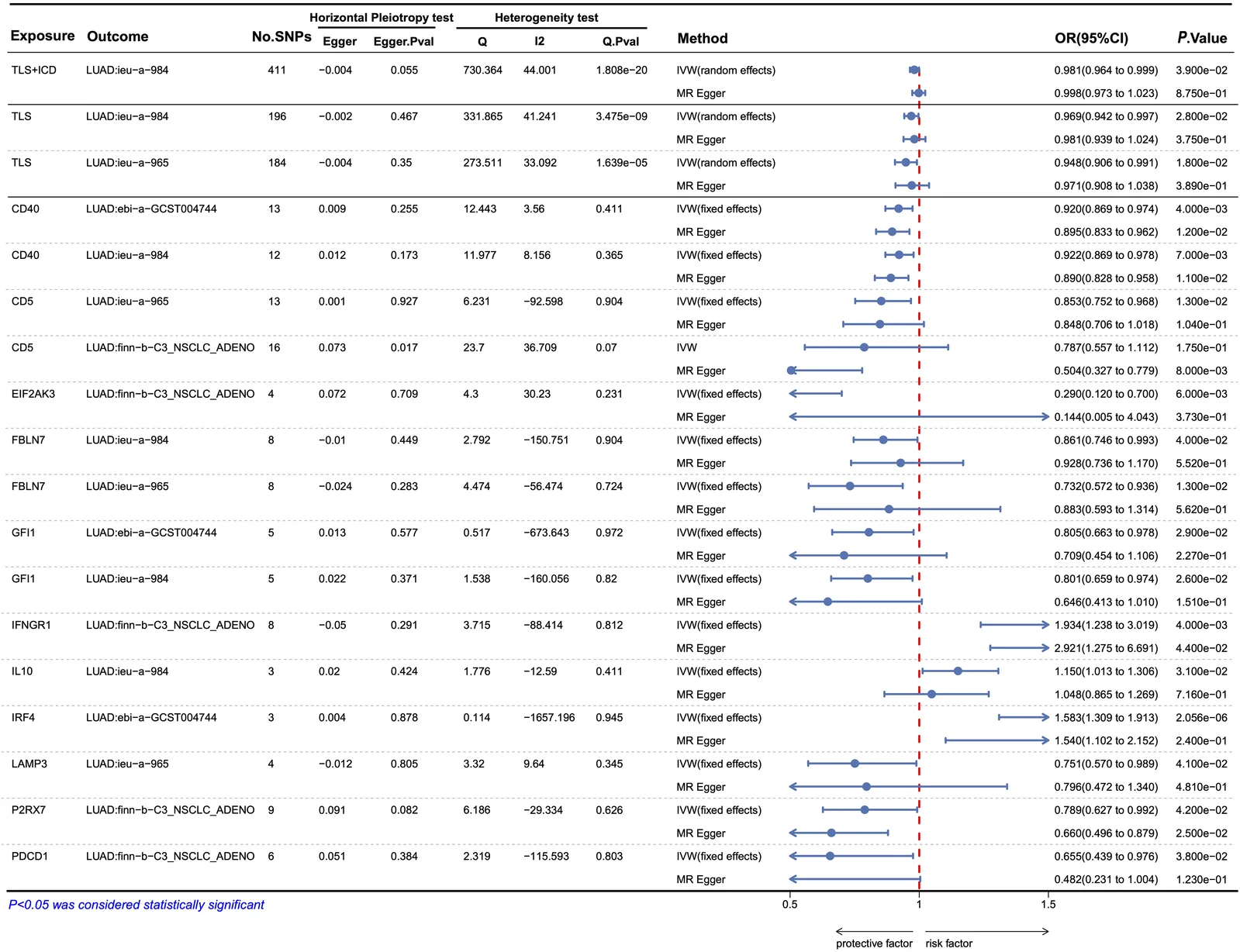
Mendelian randomization analysis of the associations between TLS-/ICD-related features and LUAD risk. Forest plot showing Mendelian randomization effect estimates for candidate TLS- and ICD-related genes in relation to LUAD. Each point represents the estimated odds ratio (OR), and horizontal bars indicate the corresponding 95% confidence intervals.

### 3.6 Validation of CD5, CD40, and GFI1 expression patterns and clinical relevance

We next focused on *CD5*, *CD40*, and *GFI1*, which were prioritized by combined survival and Mendelian randomization analyses. Initial validation using Human Protein Atlas data confirmed protein-level expression patterns for multiple TLS- and ICD-related genes, including nine TLS genes and 16 ICD genes, with staining intensity distributions summarized in **Supplementary Figure 13**.

The prognostic relevance of CD5, CD40, and GFI1 was further confirmed in the GSE37745, GSE68465, and GSE72094 cohorts, which consistently supported their expression-outcome relationships (**Supplementary Figures 14A, B**). To define their cellular origins, we systematically profiled these genes across multiple LUAD single-cell RNA-seq datasets. CD5 was consistently enriched in T-cell subsets, including CD4 Tconv, Treg, proliferating T cells, and CD8 T/CD8 Tex populations (**Figure 7A**). CD40 was mainly expressed in myeloid immune populations, particularly dendritic cells and monocyte/macrophage subsets (**Figure 7B**). In contrast, GFI1 showed lower overall expression and was primarily localized to lymphoid lineages, including T cells and NK cells (**Figure 7C**).

**Figure 7:**
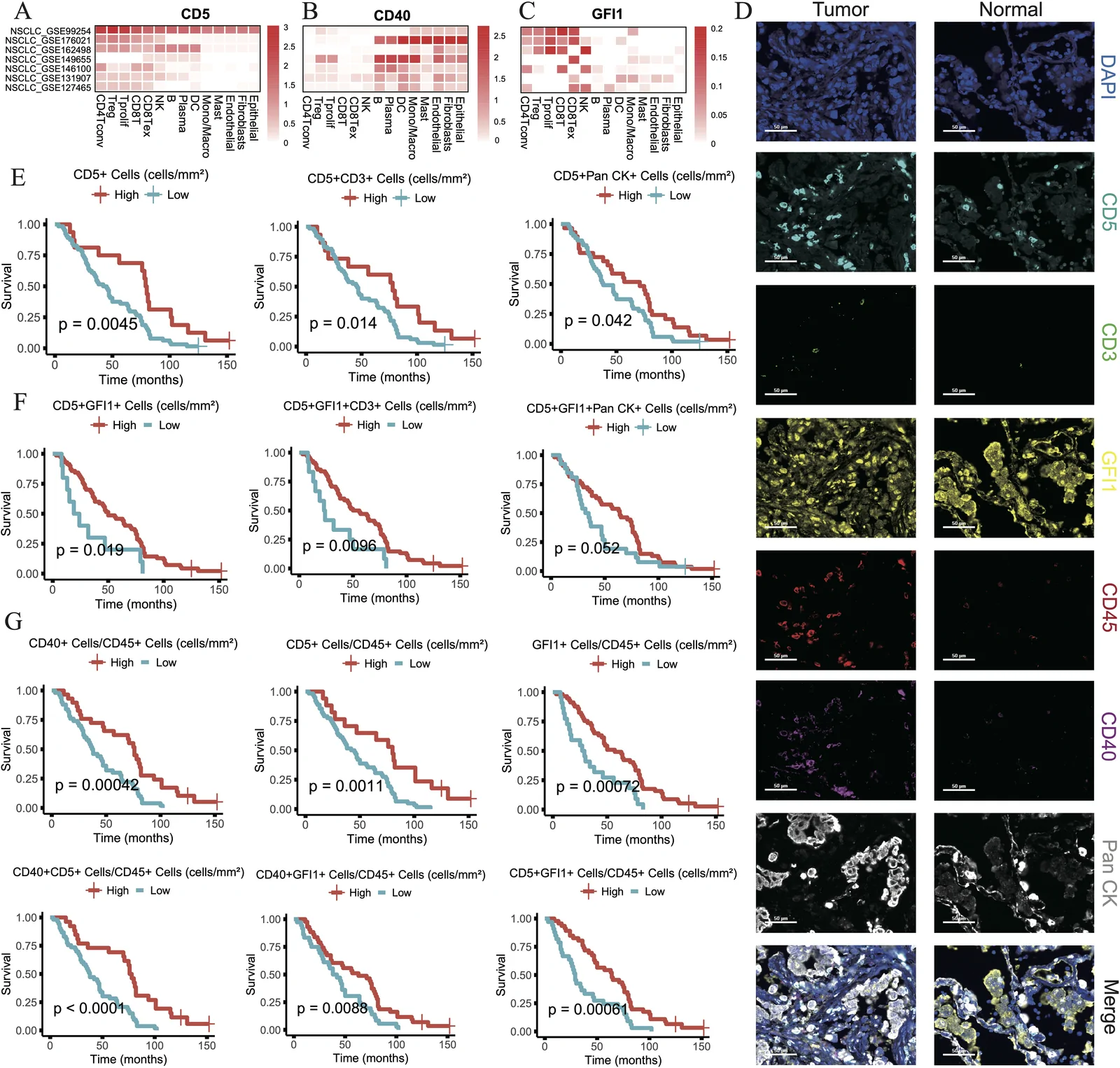
Multilevel validation of CD5, CD40, and GFI1 expression patterns and prognostic relevance in LUAD. **(A–C)** Heatmaps showing the single-cell expression patterns of **(A)** CD5, **(B)** CD40, and **(C)** GFI1 across major cell populations in multiple LUAD scRNA-seq datasets. **(D)** Representative TSA-based multiplex immunofluorescence/multiplex immunohistochemistry images of paired normal and tumor tissues showing DAPI, CD5, CD3, GFI1, CD45, CD40, PanCK, and merged signals. Scale bars, 50 μm. Kaplan–Meier curves showing the associations of **(E)** absolute densities of CD5-related phenotypes and **(F)** absolute densities of CD5–GFI1-related phenotypes with overall survival. **(G)** Kaplan–Meier curves of relative expression/compositional ratios within the CD45+ immune compartment, including CD40+/CD45+, CD5+/CD45+, GFI1+/CD45+, CD40+CD5+/CD45+, CD40+GFI1+/CD45+, and CD5+GFI1+/CD45+. Patients were dichotomized according to the optimal cutoff value for each metric.

We then performed TSA-based multiplex immunohistochemistry on paired normal and LUAD tissue microarrays to validate tissue-level phenotypes (**Figure 7D**). Notably, the populations of CD40+, CD40+CD3+, CD40+CD45+, and CD40+PanCK+ cells were significantly elevated in LUAD tissues (**Supplementary Figure 15A**). Furthermore, the population of CD5+, CD5+CD3+, and CD5+CD45+ cells was also increased in tumor tissues (**Supplementary Figure 15B**). Additionally, the signals for GFI1+CD45+ and GFI1+PanCK+ cells were markedly enhanced in the tumor samples (**Supplementary Figure 15C**). At the composite-phenotype level, there was a significant enrichment of CD40+CD5+, CD40+GFI1+, and CD5+GFI1+CD45+ cells within the tumors (**Supplementary Figures 15D–F**).

Survival analyses of absolute abundance or density metrics further demonstrated that several multiplex phenotypes were significantly associated with OS. CD40+ and CD40+CD45+ were associated with worse survival, whereas CD5+, CD5+CD3+, and CD5+PanCK+ were associated with favorable survival (**Figure 7E**; **Supplementary Figures 16A, B**). GFI1+CD45+, GFI1+PanCK+, and several CD40-GFI1-related phenotypes were associated with poorer outcomes, whereas CD5+GFI1+ and CD5+GFI1+CD3+ were associated with improved survival (**Figure 7F**; **Supplementary Figures 16C–F**).

We additionally evaluated relative expression ratios. CD40+/CD3+, CD40+/CD45+, and CD40+/PanCK+ ratios were significantly increased in tumors, whereas CD5+/CD3+, GFI1+/CD45+, and GFI1+/PanCK+ ratios were higher in normal tissues (**Supplementary Figures 17A–C**). Several composite ratios also differed significantly between tumor and normal tissues (**Supplementary Figures 17D–F**). Survival analysis of these ratio metrics further showed that multiple relative-expression features were associated with OS, including both risk-associated and protective patterns depending on the immune or epithelial reference compartment (**Figure 7G**; **Supplementary Figures 18A–F**). Together, these results support context-dependent roles of CD5, CD40, and GFI1 within the LUAD immune microenvironment.

### 3.7 Drug prediction, molecular docking and molecular dynamics analysis

To explore potential therapeutic vulnerabilities associated with the HH-risk state, we used the oncoPredict framework to estimate drug sensitivity based on genes differentially expressed between HH-risk and LL-risk tumors (**Supplementary Figure 19A**). Three compounds from the GDSC database and 20 compounds from the CTRP database were predicted to suppress genes upregulated in HH-risk tumors and/or reverse the downregulated expression patterns observed in this subgroup (**Supplementary Figure 19B**). Except for Acetalax, all 23 compounds showed significant differences in predicted IC50 values between HH-risk and LL-risk tumors, with generally higher IC50 values in LL-risk tumors, suggesting that these agents may be more effective in HH-risk tumors (**Supplementary Figure 19B**).

We next performed molecular docking analyses to assess potential interactions between candidate compounds and the key targets CD5, CD40, and GFI1. The chemical structures of the 23 compounds were retrieved from PubChem or ZINC15, and the protein structures of CD5, CD40, and GFI1 were obtained from UniProt. Molecular docking supported plausible interactions between candidate compounds and CD5, CD40, or GFI1. For CD5, BRD-K02492147, CAY10576, and SCH772984 showed the most favorable docking scores. For CD40, the top-ranked compounds were SCH772984, SZ4TA2, and CAY10576. For GFI1, SZ4TA2, SCH772984, and CAY10576 showed the best docking performance (**Figure 8B**; **Supplementary Table 15**).

**Figure 8:**
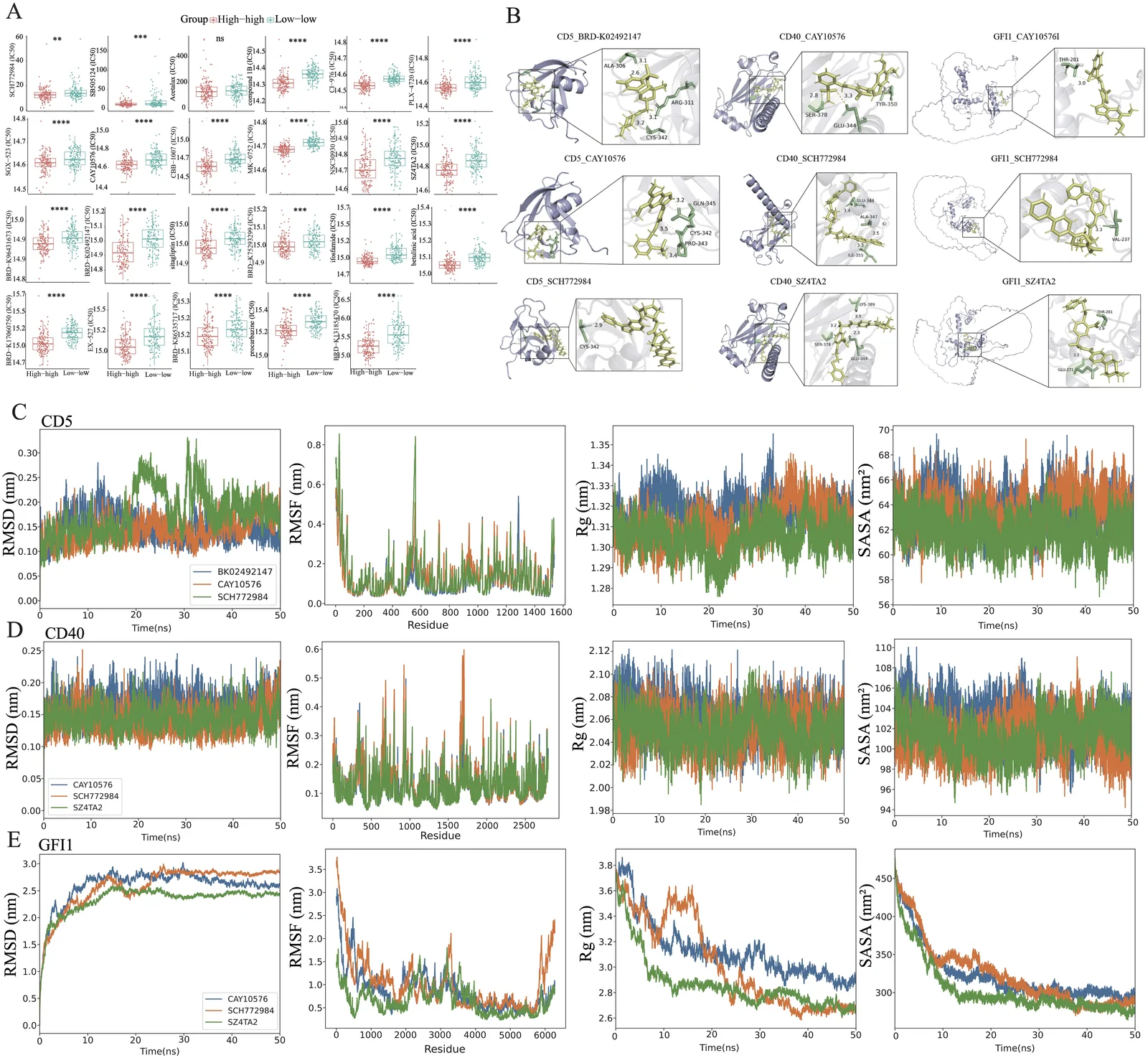
Drug sensitivity prediction, molecular docking, and molecular dynamics analysis of candidate compounds in LUAD. **(A)** Comparison of predicted IC50 values of candidate compounds between the HH-risk and LL-risk groups. **(B)** Representative molecular docking models showing the predicted binding modes of selected compounds with the key target proteins CD5, CD40, and GFI1. **(C–E)** Molecular dynamics simulation profiles of selected docked complexes for **(C)** CD5, **(D)** CD40, and **(E)** GFI1. Structural stability and dynamic behavior were evaluated using root mean square deviation (RMSD), root mean square fluctuation (RMSF), radius of gyration (Rg), and solvent-accessible surface area (SASA).

Molecular dynamics simulations further showed that the CD5- and CD40-related complexes maintained relatively stable RMSD, RMSF, Rg, and SASA profiles throughout the simulation period, supporting the stability of these ligand-protein interactions (**Figures 8C, D**). Although the GFI1-related complexes displayed greater conformational fluctuations during the early phase of the simulation, their trajectories gradually converged, accompanied by reductions in Rg and SASA, suggesting progressive conformational adaptation toward a more compact bound state (**Figure 8E**). Collectively, these findings provide preliminary computational support for the therapeutic relevance of the identified candidate compounds in high-risk LUAD.

### 3.8 Multilevel reconstruction of TLS- and ICD-related gene regulatory networks

To investigate how TLS- and ICD-related genes interact within the LUAD microenvironment, we reconstructed multilevel gene regulatory networks (GRNs) in normal and tumor tissues. We first calculated the expression index (EI), defined as the sum of the expression values of all input TLS- and ICD-related genes in each sample. Power-law fitting showed that the EI could adequately capture the relationship between global transcriptional context and individual gene expression in both normal and tumor tissues (**Supplementary Figure 20**). While many genes increased with EI in both conditions, the slopes of these relationships differed across genes and tissue states. For example, *CALR*, *ATG5*, and *PDIA3* showed similar EI-dependent trends in normal and tumor tissues, whereas CXCL13 showed a steeper slope in tumors, suggesting altered regulatory dynamics during tumorigenesis (**Supplementary Figure 20**).

To further characterize crosstalk among TLS- and ICD-related genes, we clustered genes into 11 modules on the basis of Bayesian information criterion optimization. These modules differed in size and trajectory pattern, with some modules showing similar behavior across normal and tumor tissues and others exhibiting marked condition-specific divergence (**Supplementary Figure 21**). On this basis, we reconstructed multilevel GRNs in normal and tumor tissues (**Figures 9A, B**). At the top layer, module-module interactions were dominated by connections involving M1 in both tissue states. However, tumor-specific interactions were evident. For instance, M1 inhibited M10 and M10 promoted M1 in tumor tissues, whereas this reciprocal pattern was not observed in the normal network (**Figure 9A**).

**Figure 9:**
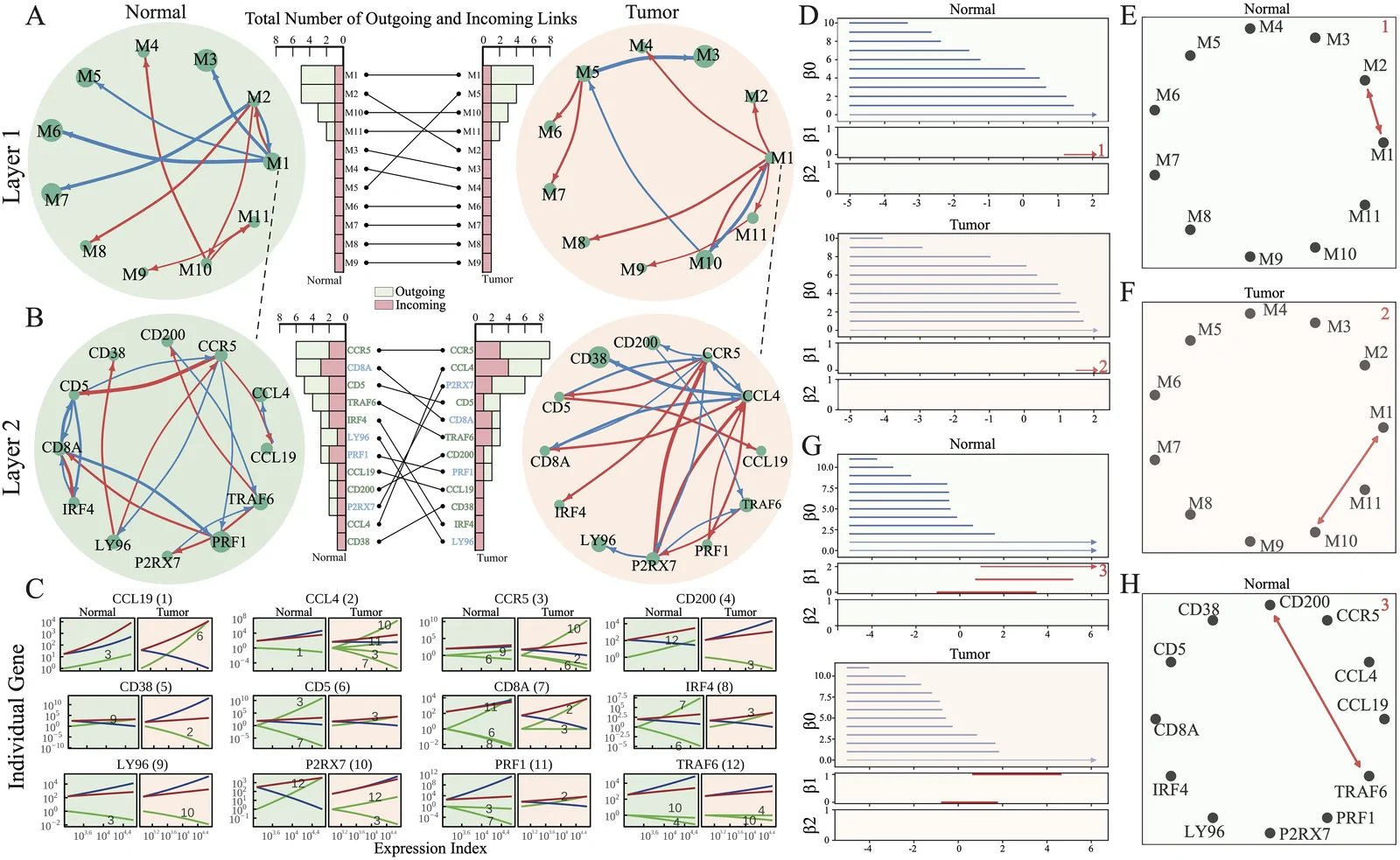
Reconstruction of multilevel gene regulatory networks and analysis of topological features in normal and LUAD tissues. **(A)** Top-layer module regulatory networks reconstructed from RNA expression data in normal and LUAD tissues. **(B)** Second-layer gene regulatory networks reconstructed for module 1 (M1) in normal and LUAD tissues. **(C)** Decomposition of the observed expression trajectories of representative genes (red) into independent components (blue) and dependent components (green) driven by regulation from other genes in the M1 network. GLMY homology-based analysis of the topological features of the **(D)** top-layer and **(G)** second-layer networks. Representative one-dimensional directed closed-loop structures identified in the **(E)** top-layer normal network, **(F)** top-layer tumor network, and **(H)** second-layer normal network.

At the gene level, GRNs were further reconstructed within each module. In module M1, *CCR5* and *CD8A* acted as hub genes in normal tissues, whereas *CCR5* and *CCL4* were the major hubs in tumor tissues (**Figure 9B**). Overall, tumor GRNs were denser than normal GRNs, indicating more extensive regulatory rewiring. Notably, *CCR5* and *CCL4* showed mutual inhibition in the tumor network, a pattern absent in the normal state. We then used the qdGGT framework to decompose gene expression into network-dependent and network-independent components (**Figure 9C**). For example, *CD200* showed similar total expression between normal and tumor tissues, but its inferred regulatory architecture differed: it was promoted by *TRAF5* in normal tissues and inhibited by *CCR5* in tumor tissues (**Figures 9B, C**). These findings indicate that transcriptionally similar genes may still occupy distinct regulatory roles depending on disease context.

### 3.9 Topological characteristics of GRNs in normal and tumor tissues

We next applied GLMY homology theory to characterize the higher-order topology of the reconstructed GRNs. At the top module layer, the initial state consisted of 11 disconnected subnetworks. As filtration progressed, directed edges were gradually incorporated, ultimately yielding a single connected component in the final state (**Figure 9D**). In dimension 1, one persistent directed closed loop was identified, whereas no dimension-2 cavity-like structures were detected in either normal or tumor tissues (**Figure 9D**).

In the normal top-layer GRN, a one-dimensional directed closed loop was formed between M1 and M2, indicating stable reciprocal regulation between these modules (**Figure 9E**). In contrast, the tumor top-layer GRN was characterized by a distinct loop involving M1 and M10 (**Figure 9F**), indicating topological reorganization of module-level regulatory architecture during tumorigenesis.

More pronounced differences were observed at the gene level. In tumor tissues, the final network state contained only one connected component and no persistent one- or two-dimensional homology groups, despite the transient formation of two closed loops during filtration (**Figure 9G**). By contrast, the normal gene-level network retained two connected components and one persistent directed loop in the final state. In particular, *CD200* and *TRAF6* formed a stable directed closed loop in the normal network (**Figure 9H**), suggesting sustained reciprocal regulation under physiological conditions. Overall, these results indicate that tumor GRNs exhibit a simplified and less persistent higher-order topology than normal GRNs, consistent with disruption of coordinated immune-regulatory organization in LUAD.

### 3.10 Topological characteristics of GRNs in HH-risk and LL-risk tumors

We further investigated condition-specific GRNs in the HH-risk and LL-risk groups. EI-based power-law fitting again showed that expression trajectories were well captured by the quasi-dynamic framework (**Supplementary Figure 22**). TLS- and ICD-related genes were grouped into eight modules according to BIC optimization (**Supplementary Figure 23**), and two-layer GRNs were reconstructed for HH-risk and LL-risk tumors (**Figures 10A, B**).

**Figure 10:**
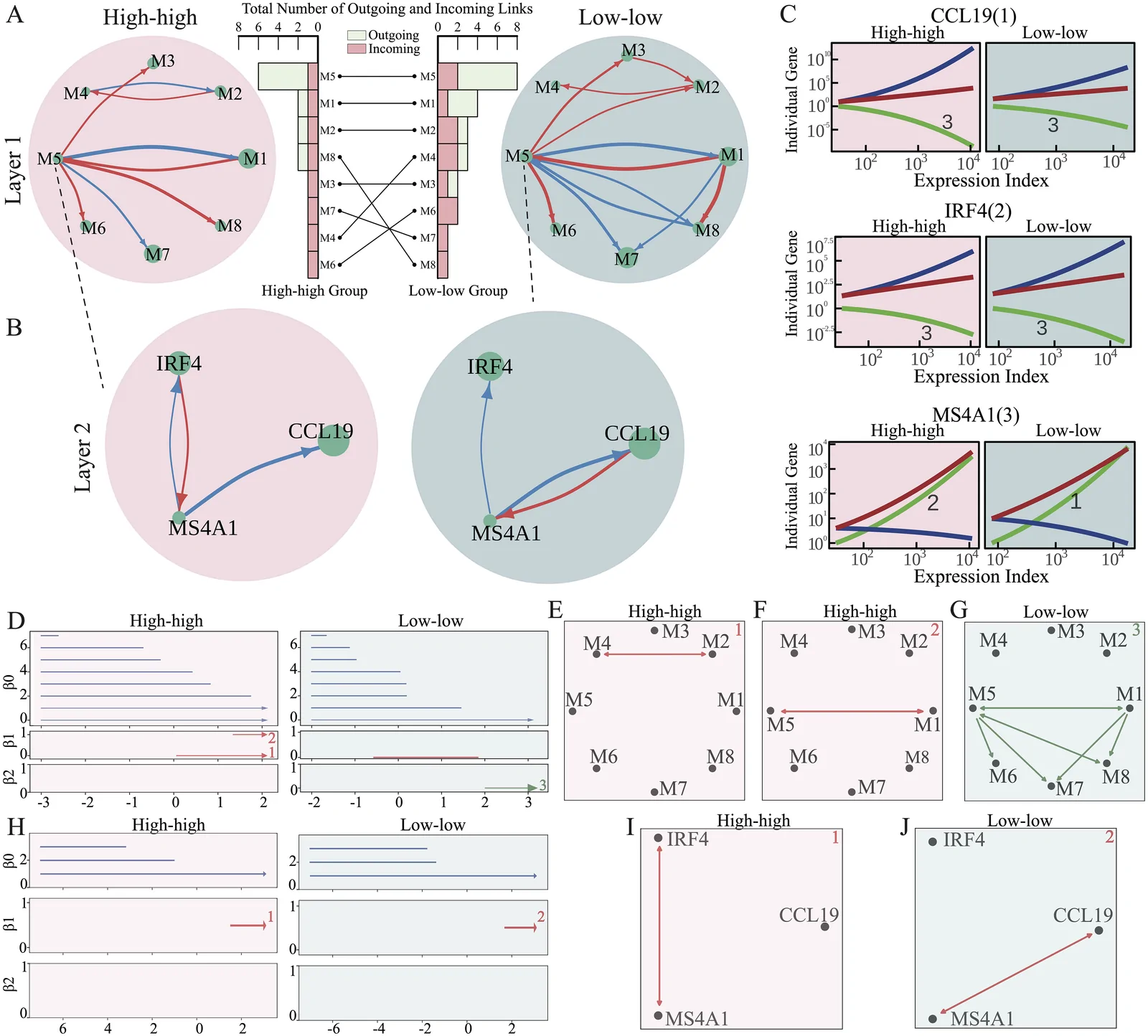
Differences in multilevel gene regulatory networks and topological features between the HH-risk and LL-risk groups. **(A)** Top-layer gene regulatory networks reconstructed from RNA expression data in the HH-risk and LL-risk groups. **(B)** Second-layer gene regulatory networks reconstructed for module 5 (M5). **(C)** Decomposition of the observed expression trajectories of representative genes (red) into independent components (blue) and dependent components (green) driven by regulation from other genes in the M5 network. GLMY homology-based analysis of the topological features of the **(D)** top-layer and **(H)** second-layer networks. Representative one-dimensional directed closed-loop structures identified in the **(E, F)** top-layer HH-risk network, **(I)** second-layer HH-risk network, and **(J)** second-layer LL-risk network. **(G)** Representative two-dimensional directed structure identified in the top-layer LL-risk network.

At the module level, the LL-risk network showed a more complex architecture than the HH-risk network. M5 was identified as the dominant module in both groups, but their interaction structures differed markedly (**Figure 10A**). At the gene level, module-specific GRNs further highlighted risk-state-dependent rewiring. For example, *MS4A1* inhibited *IRF4* and *CCL19* in both HH-risk and LL-risk tumors, whereas the feedback direction differed: *IRF4* promoted *MS4A1* in HH-risk tumors, while *CCL19* promoted *MS4A1* in LL-risk tumors (**Figure 10B**). Moreover, the independent effect of *CCL19* was greater in HH-risk tumors, whereas the independent effect of *IRF4* was greater in LL-risk tumors, despite similar bulk expression levels (**Figure 10C**). These results suggest that HH-risk and LL-risk tumors differ not only in expression profiles but also in underlying regulatory logic.

GLMY analysis further revealed distinct topological organizations between the two risk states. At the top layer, the HH-risk network retained two connected components and two persistent one-dimensional directed loops, whereas the LL-risk network showed a single connected component and no final one-dimensional loop (**Figure 10D**). Specifically, M2-M4 and M1-M5 formed persistent directed loops in the HH-risk network (**Figures 10E, F**). In contrast, the LL-risk top-layer network contained a two-dimensional cavity-like structure involving M1, M5, M6, M7, and M8 (**Figure 10G**), indicating the presence of higher-order regulatory organization not observed in HH-risk tumors. These findings suggest that HH-risk tumors retain more persistent low-dimensional regulatory loops, whereas LL-risk tumors exhibit more complex higher-order immune-regulatory topology.

At the gene layer, the topological patterns of HH-risk and LL-risk networks were more similar overall (**Figure 10H**), but the core one-dimensional loops differed. In HH-risk tumors, a directed closed loop was formed between *MS4A1* and *IRF4* (**Figure 10I**), whereas in LL-risk tumors the corresponding stable loop involved *MS4A1* and *CCL19* (**Figure 10J**). Together, these results indicate that distinct risk states in LUAD are accompanied by marked rewiring of both regulatory interactions and higher-order network topology.

## 4 Discussion

ICD and TLSs represent two biologically distinct but closely connected dimensions of antitumor immunity in LUAD. ICD-associated genes primarily reflect cellular stress, regulated cell death, and immunogenic signaling, highlighting the capacity of dying tumor cells to initiate immune activation through danger-associated signals such as DAMPs (14, 92, 93). By contrast, TLS-related genes reflect the local immune organization required for antigen presentation, lymphocyte priming, and adaptive immune amplification within or adjacent to tumors (94). When considered independently, ICD-related programs may capture the ability of tumor cells to generate immunostimulatory signals, whereas TLS-related programs more directly reflect whether these signals are embedded within an immune context capable of sustaining effective local immune activation (94, 95). From this perspective, integrating ICD- and TLS-related programs provides a more comprehensive view of antitumor immunity in LUAD, spanning immunogenic signal release, immune-cell recruitment, local immune organization, and downstream effector responses.

Although both TLSs and ICD are generally associated with favorable antitumor immunity, their biological significance is likely to be context-dependent rather than uniformly protective. In the present study, the HH-risk subgroup does not indicate high productive TLS activity or high effective ICD activity; rather, it denotes patients with both high TLS-related and high ICD-related prognostic risk scores. Therefore, the inferior survival of HH-risk patients may reflect a dysregulated immune state in which immune-related transcriptional signals are present but fail to translate into coordinated and effective antitumor immunity. Such a state may arise from immature or dysfunctional TLSs, chronic inflammatory activation without efficient immune priming, immune exhaustion, or immunosuppressive remodeling (96, 97). In contrast, the LL-risk state may represent a more coordinated immune context in which ICD-associated immunogenic signals are embedded within TLS-supported antigen presentation, lymphocyte activation, and adaptive immune amplification. These observations suggest that the prognostic impact of TLS- and ICD-related programs depends not simply on their abundance, but on whether they are coordinately maintained within an immune-supportive microenvironment (64, 98, 99).

Our Mendelian randomization and survival analyses converged on three candidate protective genes, *CD40*, *CD5*, and *GFI1*, all of which were consistently downregulated in LUAD and in the HH-risk subgroup. These genes are functionally linked to immune regulation and may represent complementary components of the TLS–ICD axis. *CD40*, a member of the TNF receptor superfamily, is highly expressed on antigen-presenting cells and promotes dendritic-cell maturation, IL-12 secretion, and CD8+ T-cell activation through CD40–CD40L signaling (100, 101). In the context of ICD, *CD40* may help convert tumor antigen release and damage-associated immune stimulation into productive antigen presentation and T-cell priming, thereby linking ICD-associated signals to TLS-supported adaptive immune activation. *CD5* is an important regulator of lymphocyte activation and immune homeostasis, particularly in T cells and B-1a cells (102, 103). Its enrichment in lymphoid populations suggests that CD5 may reflect the organization and functional tuning of tumor-infiltrating lymphocytes within TLS-like immune niches. *GFI1* is a zinc-finger transcriptional repressor involved in hematopoiesis, immune-cell differentiation, neutrophil development, and inflammatory restraint (104, 105). Thus, *GFI1* may contribute to immune-lineage balance and the control of inflammatory remodeling within the tumor microenvironment. Taken together, *CD40*, *CD5*, and *GFI1* may support effective immune surveillance by connecting antigen presentation, lymphocyte organization, and immune-cell differentiation. Their suppression in LUAD and in the HH-risk state may therefore indicate impaired conversion of ICD-associated immune stimulation into coordinated TLS-supported antitumor immunity, thereby contributing to immune escape and disease progression.

An additional finding of our study was the cross-layer discordance between transcriptomic and spatial protein-level readouts for CD40, CD5, and GFI1. This should not be interpreted as a contradiction, but rather as a reflection of the different biological resolutions captured by each data type. Bulk transcriptomics provides tissue-averaged signals and is strongly influenced by tumor purity and cellular composition, whereas TSA-based multiplex immunohistochemistry resolves marker expression and co-localization at the single-cell and tissue-niche levels. In this context, the enrichment of CD40-related, CD5-related, and composite phenotypes such as CD40+CD5+ and CD40+GFI1+ likely reflects coordinated remodeling of antigen-presentation, lymphocyte-infiltration, and immune–epithelial interaction programs rather than simple marker upregulation (106–111). Importantly, survival associations were highly context-dependent: CD5 generally remained protective, whereas the absolute abundance of several CD40- and GFI1-related phenotypes was more often associated with adverse outcome. These observations suggest that CD40, CD5, and GFI1 are better interpreted as context-dependent structural readouts of the LUAD immune ecosystem than as uniformly favorable or unfavorable biomarkers. In particular, ratio-based and composite phenotypes may capture prognostically relevant immune-ecology remodeling more robustly than absolute densities.

Our drug prediction, molecular docking, and molecular dynamics analyses further suggested that high-risk LUAD states may harbor distinct therapeutic vulnerabilities. Among the prioritized compounds, CAY10576, SCH772984, and SZ4TA2 were of particular interest. CAY10576 is a selective IKKϵ inhibitor, and aberrant IKKϵ/IKBKE activity has been linked to tumor-promoting inflammatory signaling, prosurvival transcriptional programs, and cross-talk with receptor tyrosine kinase pathways in cancer, including EGFR-related biology in NSCLC (112–114). SCH772984 is a potent ERK1/2 inhibitor that suppresses both ERK catalytic activity and ERK phosphorylation, thereby sustaining inhibition of MAPK signaling, a key pathway in tumor-cell proliferation, survival, and adaptive resistance (115–118). SZ4TA2 functions as a Bcl-xL/BH3 interaction modulator and may lower the apoptotic threshold of tumor cells, consistent with the known role of Bcl-xL as an antiapoptotic dependency in lung cancer (119–121). By comparison, BRD-K02492147 should currently be regarded as hypothesis-generating. Collectively, these findings suggest that dysregulated high-risk LUAD states may be associated with targetable vulnerabilities involving inflammatory-survival signaling, mitogenic MAPK signaling, and antiapoptotic buffering.

The multilayer gene regulatory network reconstruction and GLMY topological analysis extend these findings from differential expression to systems-level immune-regulatory organization. From normal to tumor tissues, and from low-risk to high-risk tumors, we observed progressive simplification of higher-order topological structures, suggesting disruption of coordinated immune-regulatory architecture. Biologically, this may reflect a transition from a relatively organized immune ecosystem toward a more fragmented and dysregulated state in which antitumor communication becomes less effectively coordinated (122). Notably, low-risk tumors retained more complex higher-order organization, whereas high-risk tumors were characterized by more restricted and loop-dominated regulatory structures, consistent with a less adaptive and more tumor-permissive immune landscape (123). These results indicate that LUAD heterogeneity is encoded not only in gene-expression abundance, but also in the higher-order organization of immune-related regulatory networks. At the same time, because these networks were inferred from cross-sectional transcriptomic data, the observed topological patterns should be interpreted as systems-level signatures of regulatory remodeling rather than direct evidence of causal dynamics.

Overall, our findings support a model in which ICD-associated immunogenic signaling and TLS-associated immune organization jointly define clinically relevant antitumor immune states in LUAD. The biological significance of ICD-associated signaling appears to depend not only on its presence, but also on whether it is embedded within a TLS-supported immune context capable of sustaining effective local immune activation. When these two programs are coordinately maintained, they are associated with more favorable immune phenotypes and clinical outcome; when they become uncoupled, they are associated with immune disorganization, adverse prognosis, and potential therapeutic vulnerability. These findings provide a cell-death-associated immune framework for understanding LUAD heterogeneity and highlight candidate biomarkers and therapeutic targets linked to coordinated disruption of TLS and ICD programs.

## 5 Limitations

This study has several limitations. First, although single-cell RNA-seq and TSA-based multiplex immunohistochemistry were incorporated as complementary validation approaches, most analyses were still based on bulk transcriptomic data and therefore could not fully resolve the cell-type-specific and spatial coordination between ICD-associated signaling and TLS-associated immune organization in LUAD. Second, because the TLS–ICD risk model was trained on bulk transcriptomic data, its application to single-cell RNA-seq data should be interpreted as an exploratory assessment of relative TLS-/ICD-related transcriptional states rather than as a direct clinical risk classification of individual cells. Third, the drug prediction, molecular docking, and molecular dynamics analyses provide preliminary support for potential therapeutic vulnerabilities, but these findings remain exploratory and require direct biological and pharmacological validation. Fourth, the multilayer network reconstruction and topological analyses provide systems-level signatures of immune-regulatory remodeling, but they should not be interpreted as direct evidence of causal dynamics. Finally, although our data support coordinated involvement of ICD- and TLS-related programs in LUAD, further functional studies will be required to define the mechanistic coupling between cell-death-associated immune activation and local immune organization.

## 6 Conclusions

In summary, this study identifies a coordinated TLS–ICD immune axis that shapes clinically relevant antitumor immune states in LUAD. Integrated analysis of TLS- and ICD-related programs distinguished high- and low-risk immune states with distinct immune infiltration patterns, tumor microenvironment features, immunotherapy-related characteristics, and clinical outcomes. CD40, CD5, and GFI1 were further implicated as context-dependent immune-regulatory genes supported by genetic, transcriptomic, single-cell, and spatial protein-level evidence. Exploratory drug prediction and structural analyses suggested potential therapeutic vulnerabilities in high-risk LUAD, while multilayer gene regulatory network reconstruction and GLMY topology analysis revealed disruption of higher-order immune-regulatory organization during tumor progression and risk-state transition. Together, these findings provide a systems-level view of how cell-death-associated immune signaling and TLS-associated immune organization jointly contribute to LUAD heterogeneity, prognosis, and therapeutic vulnerability.

## Data Availability

The datasets presented in this study can be found in online repositories. The names of the repository/repositories and accession number(s) can be found in the article/**Supplementary Material**.
