# Supplementary material for: Immunogenic cell death and tertiary lymphoid structures jointly define dysregulated antitumor immune states and therapeutic vulnerability in lung adenocarcinoma
Source: Front Immunol. 2026 Sep 30;17:1917959. doi: 10.3389/fimmu.2026.1917959 (PMC13662155; doi:10.3389/fimmu.2026.1917959)
Supplement: Supplementary file 1 [file DataSheet1.pdf]

Supplementary figures and legends

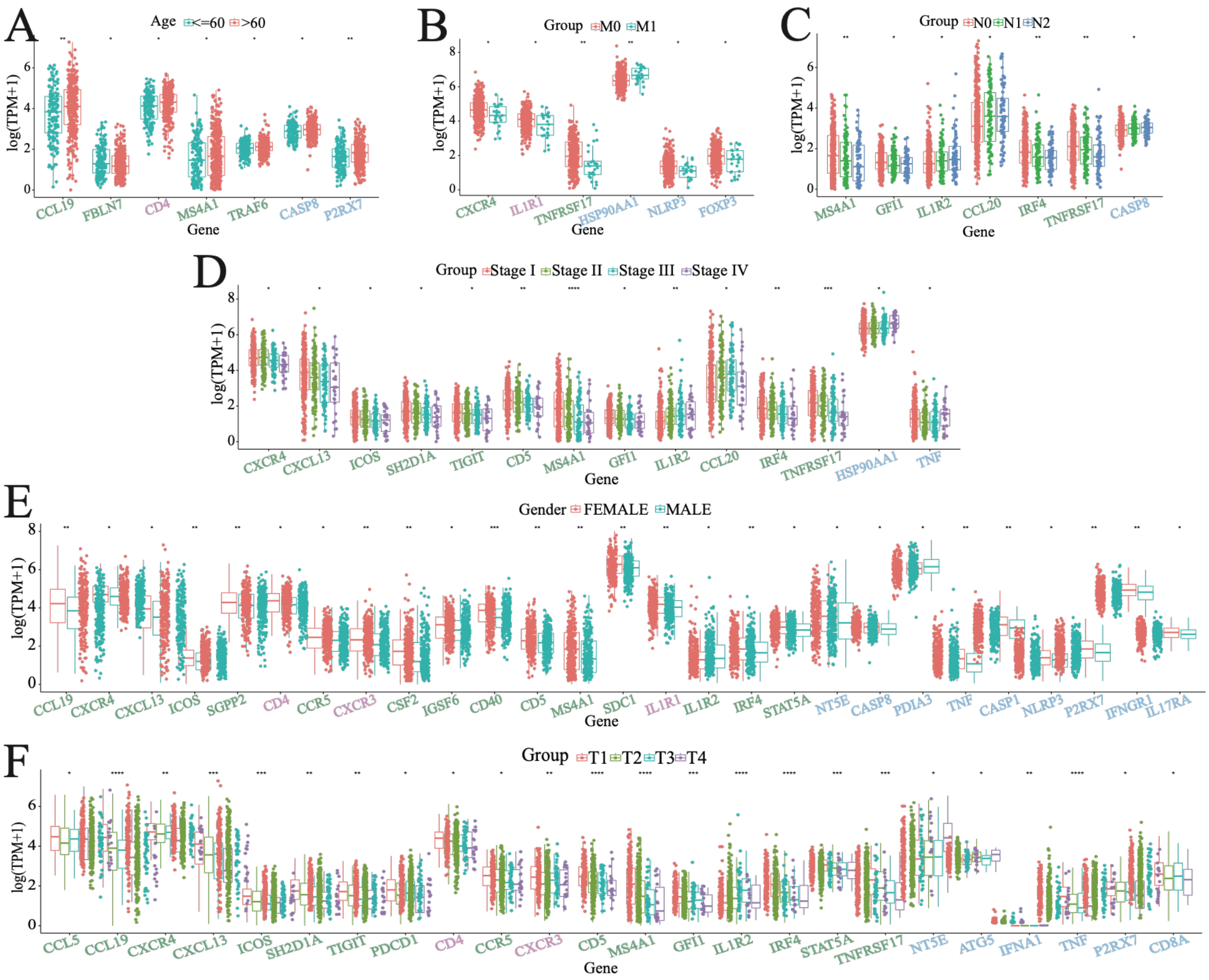

Supplementary Figure 1. Differential expression of TLS- and ICD-related genes across clinical subgroups of patients with LUAD: (A) age, (B) M stage, (C) N stage, (D) overall stage, (E) sex, and (F) T stage.

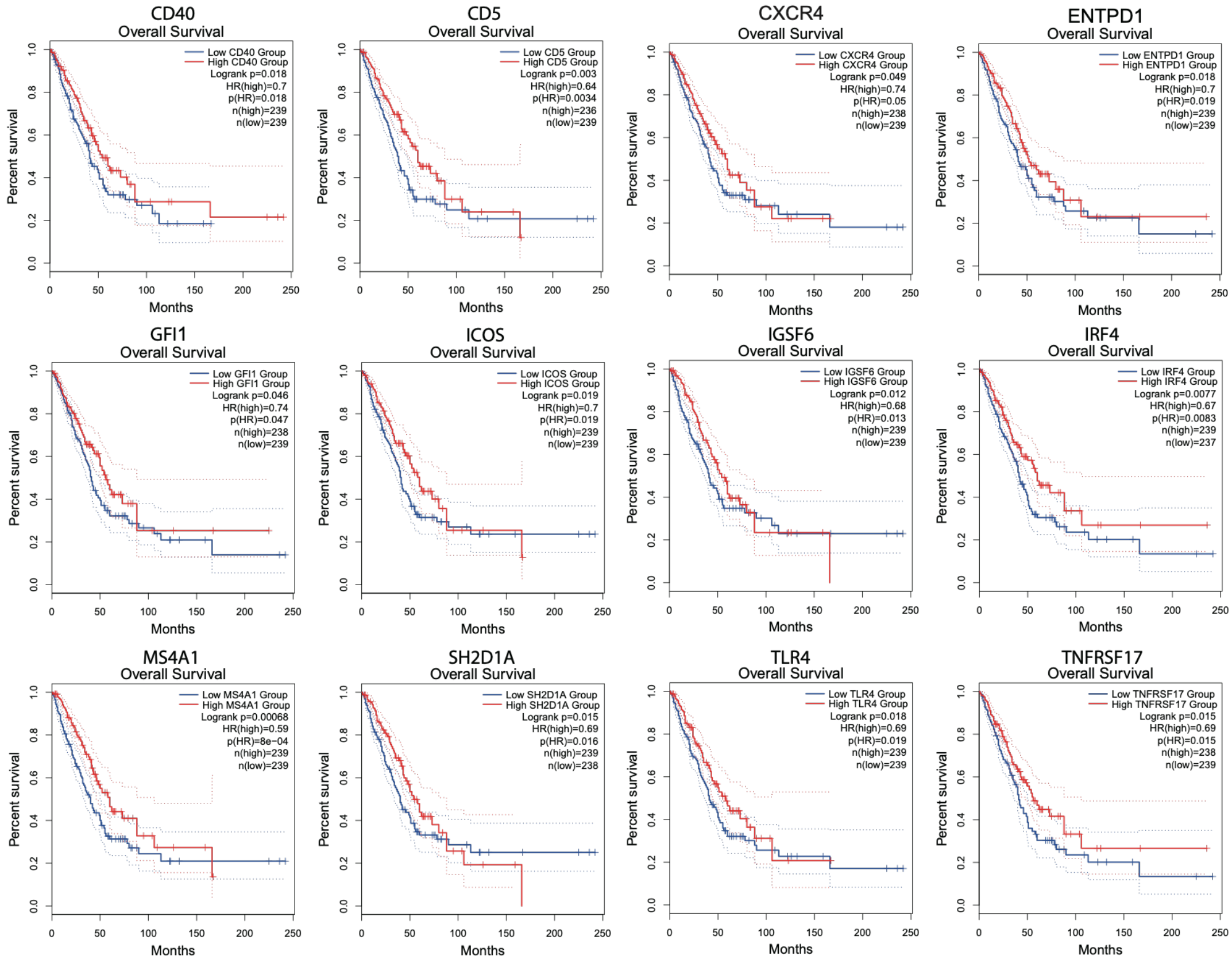

Supplementary Figure 2. Kaplan–Meier survival curves of protective TLS- and ICD-related genes associated with overall survival (OS) in patients with LUAD.

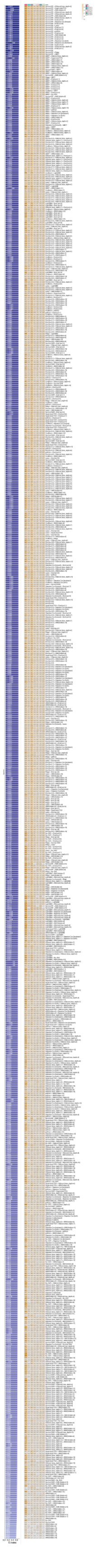

Supplementary Figure 3. Construction and validation of TLS-derived prognostic signatures using the integrative 801-machine-learning-model framework.

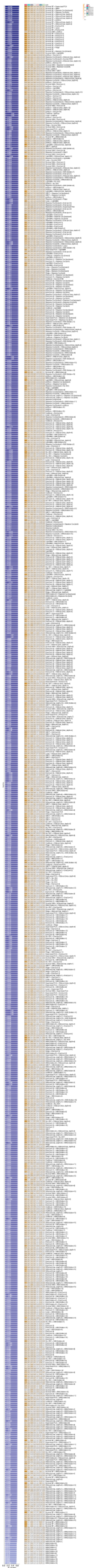

Supplementary Figure 4. Construction and validation of ICD-derived prognostic signatures using the integrative 801-machine-learning-model framework.

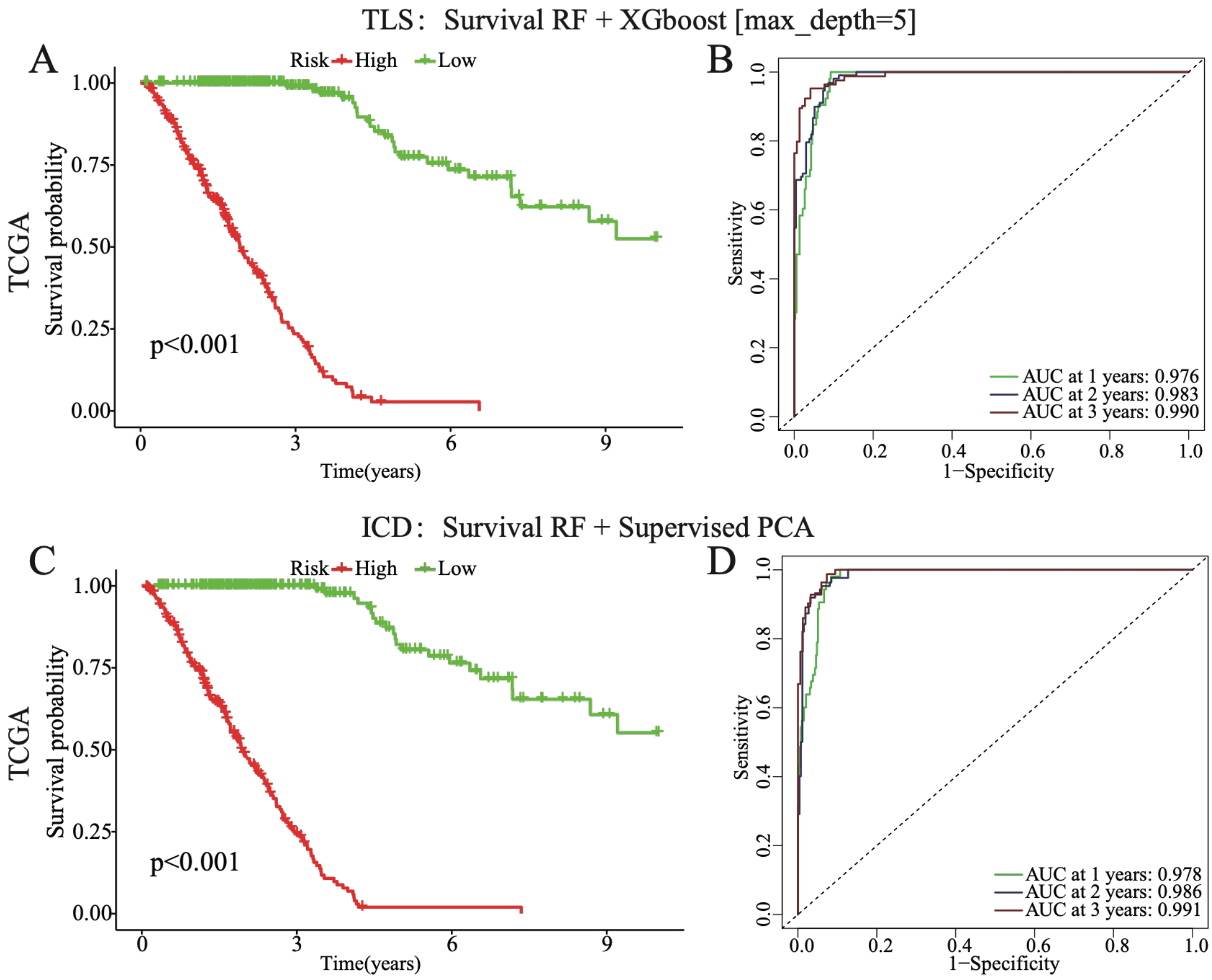

Supplementary Figure 5. Kaplan–Meier survival curves comparing (A) high- and low-risk groups based on the TLS model and (C) high- and low-risk groups based on the ICD model. ROC curves showing the AUC values of the (B) TLS model and (D) ICD model at different survival time points.

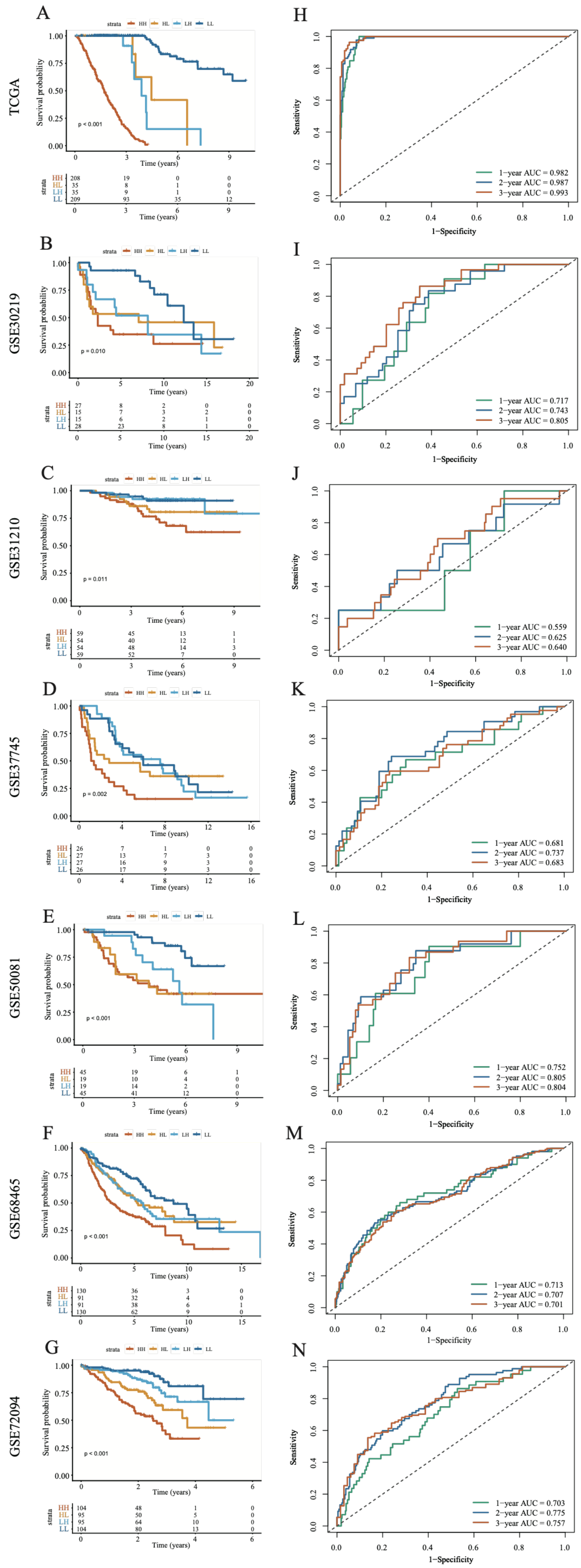
A
TCGA
strata HH HL LH LL
Survival probability
p < 0.001
Time (years)
HH 208 19 0 0
HL 35 8 1 0
LH 35 9 1 0
LL 209 93 35 12
H
Sensitivity
1−Specificity
1−year AUC = 0.982
2−year AUC = 0.987
3−year AUC = 0.993
B
GSE30219
p = 0.010
HH 27 8 2 0 0
HL 15 7 3 2 0
LH 15 6 2 1 0
LL 28 23 8 1 0
I
1−year AUC = 0.717
2−year AUC = 0.743
3−year AUC = 0.805
C
GSE31210
p = 0.011
HH 59 45 13 1
HL 54 40 12 1
LH 54 48 14 3
LL 59 52 7 0
J
1−year AUC = 0.559
2−year AUC = 0.625
3−year AUC = 0.640
D
GSE37745
p = 0.002
HH 26 7 1 0 0
HL 27 13 7 3 0
LH 27 16 9 3 0
LL 26 17 7 3 0
K
1−year AUC = 0.681
2−year AUC = 0.737
3−year AUC = 0.683
E
GSE50081
p < 0.001
HH 45 19 6 1
HL 19 10 4 0
LH 19 14 2 0
LL 45 41 12 0
L
1−year AUC = 0.752
2−year AUC = 0.805
3−year AUC = 0.804
F
GSE68465
p < 0.001
HH 130 36 3 0
HL 91 32 4 0
LH 91 38 6 1
LL 130 62 9 0
M
1−year AUC = 0.713
2−year AUC = 0.707
3−year AUC = 0.701
G
GSE72094
p < 0.001
HH 104 48 1 0
HL 95 50 5 0
LH 95 64 10 0
LL 104 80 13 0
N
1−year AUC = 0.703
2−year AUC = 0.775
3−year AUC = 0.757

Supplementary Figure 6. Prognostic performance of the integrated TLS–ICD risk-state classification across TCGA and external validation cohorts. (A–G) Kaplan–Meier survival curves comparing the four integrated TLS–ICD risk states, including high TLS-risk/high ICD-risk (HH-risk), high TLS-risk/low ICD-risk (HL-risk), low TLS-risk/high ICD-risk (LH-risk), and low TLS-risk/low ICD-risk (LL-risk), in the TCGA cohort and six independent GEO validation cohorts: GSE30219, GSE31210, GSE37745, GSE50081, GSE68465, and GSE72094. (H–N) Time-dependent ROC curves evaluating the predictive performance of the integrated TLS–ICD risk-state classification for 1-, 2-, and 3-year overall survival in the corresponding cohorts.

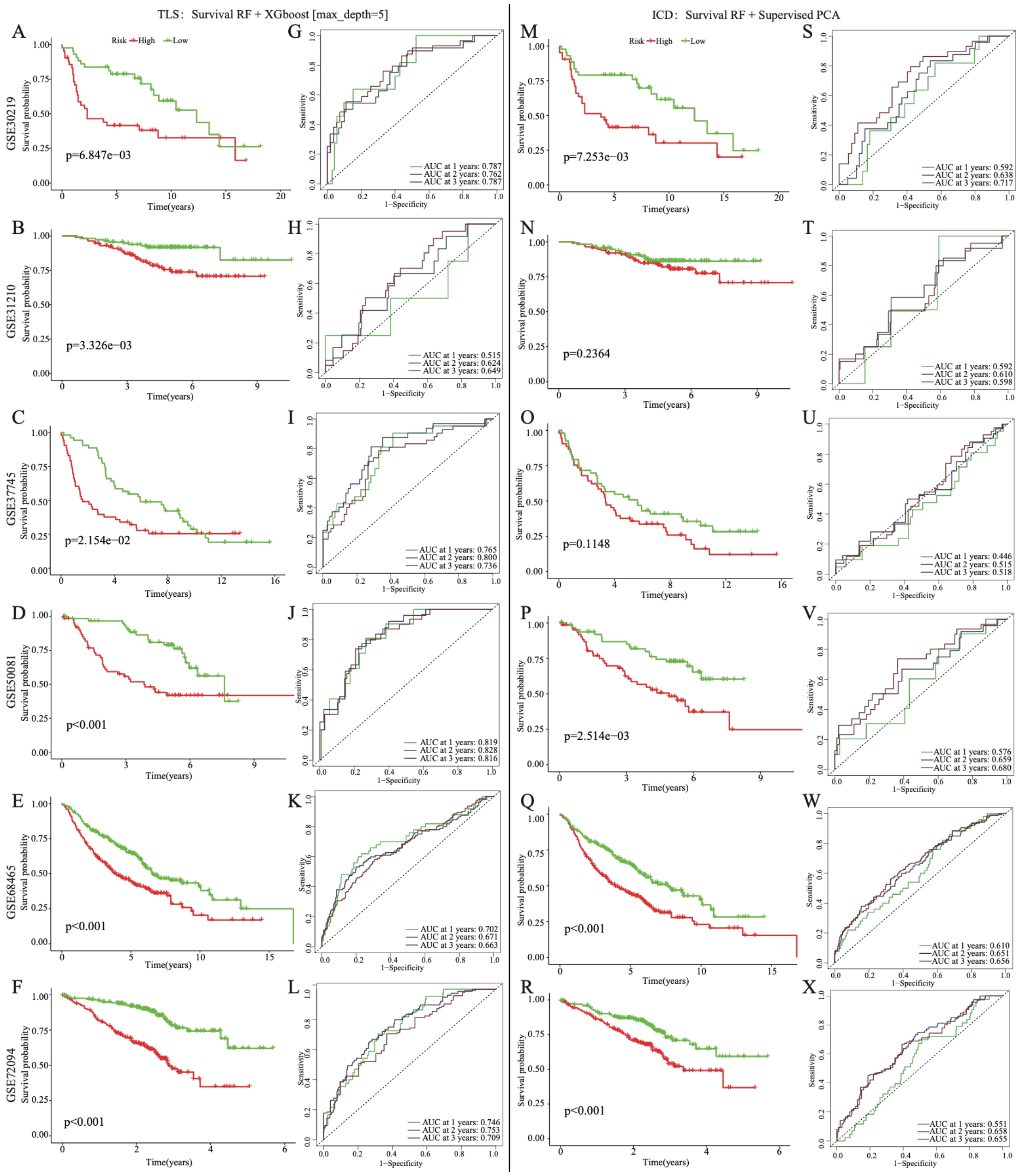

Supplementary Figure 7. Survival and ROC analyses of the TLS and ICD models in six GEO cohorts. (A–F) Kaplan–Meier survival curves based on the TLS model in six GEO datasets. (G–L) ROC curves showing the AUC values of the TLS model at different survival time points in the six GEO datasets. (M–R) Kaplan–Meier survival curves based on the ICD model in the six GEO datasets. (S–X) ROC curves showing the AUC values of the ICD model at different survival time points in the six GEO datasets.

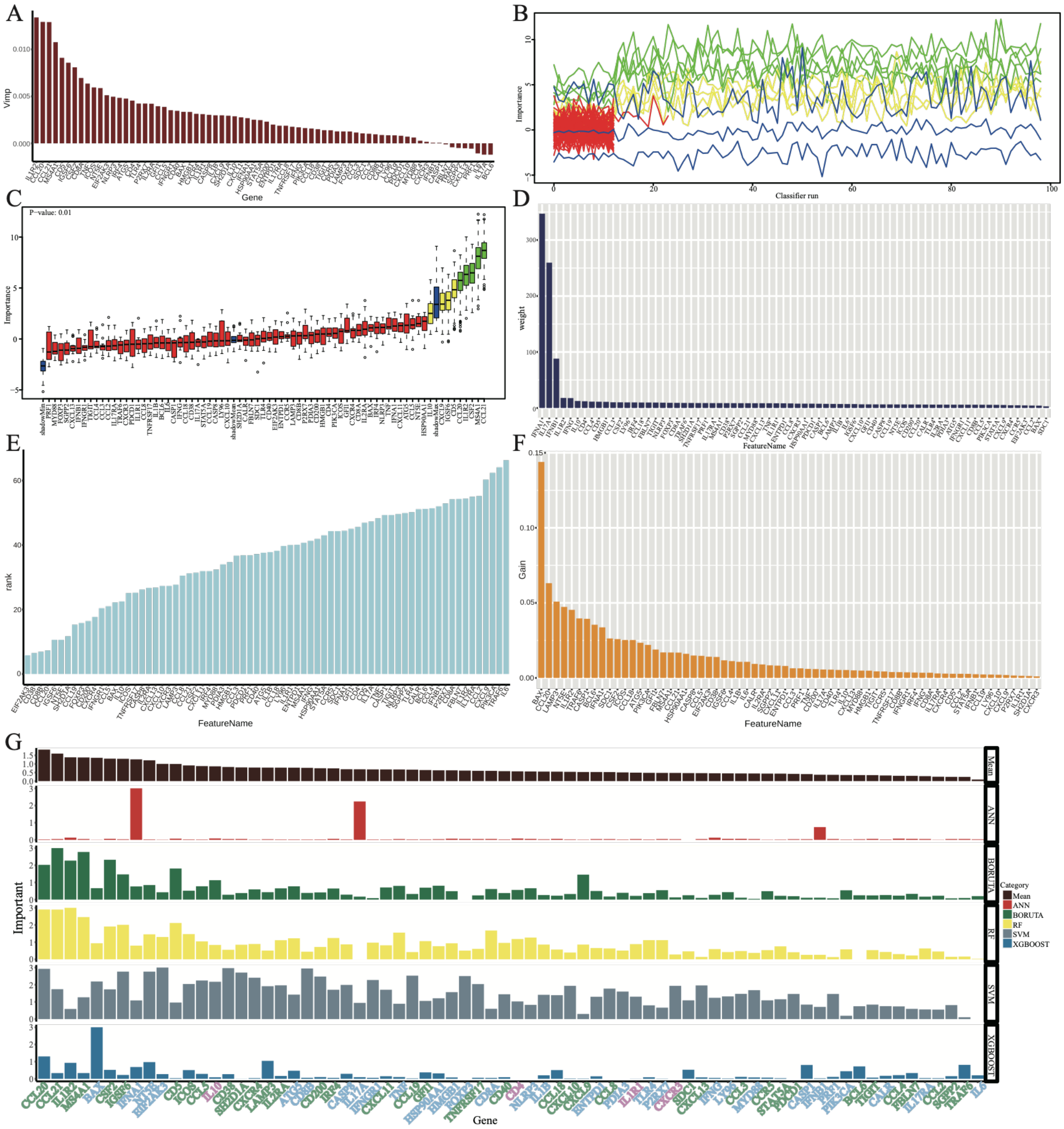

Supplementary Figure 8. Prediction of important prognostic genes for survival using five machine-learning methods. (A) Random forest (RF). (B–C) Boruta. (D) artificial neural network (ANN). (E) support vector machine (SVM). (F) XGBoost. (G) Z-score–scaled importance values of genes for predicting the prognosis of patients with LUAD across the five machine-learning methods.

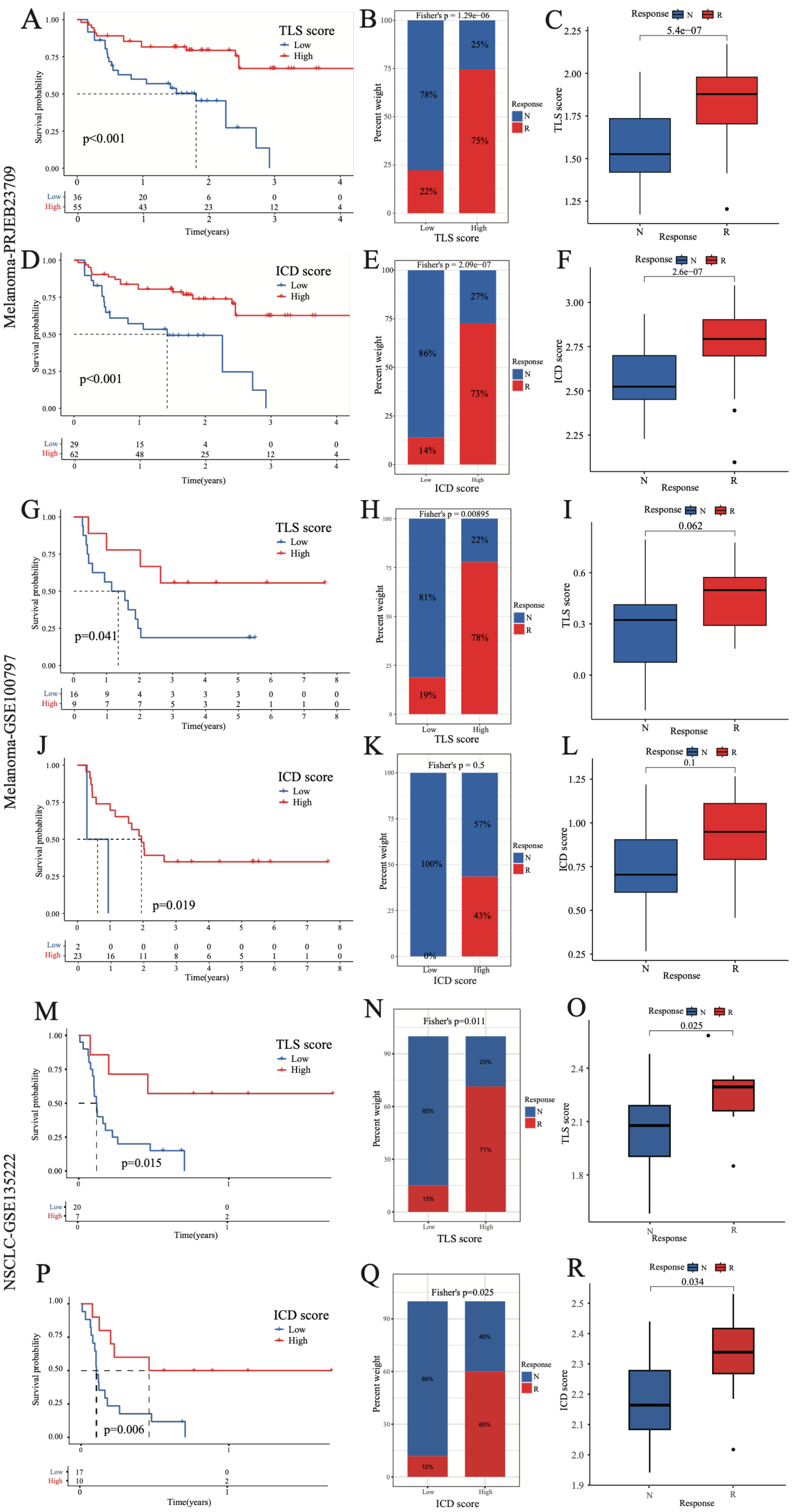
Melanoma-PRJEB23709
Melanoma-GSE100797
NSCLC-GSE135222
A
TLS score
Low
High
p<0.001
Survival probability
Time(years)
B
Fisher's p = 1.29e−06
78%
22%
25%
75%
Percent weight
TLS score
Response
N
R
C
5.4e−07
TLS score
Response
D
ICD score
p<0.001
E
Fisher's p = 2.09e−07
86%
14%
27%
73%
ICD score
F
2.6e−07
ICD score
G
TLS score
p=0.041
H
Fisher's p = 0.00895
81%
19%
22%
78%
I
0.062
J
ICD score
p=0.019
K
Fisher's p = 0.5
100%
0%
57%
43%
L
0.1
M
TLS score
p=0.015
N
Fisher's p=0.011
85%
15%
29%
71%
O
0.025
P
ICD score
p=0.006
Q
Fisher's p=0.025
88%
12%
40%
60%
R
0.034

Supplementary Figure 9. Analysis of immunotherapy-treated patients. Kaplan–Meier survival curves showing the relationship between OS and TLS or ICD scores in patients receiving immunotherapy from (A, D) PRJEB23709, (G, J) GSE100797, and (M, P) GSE135222. Proportions of responders and nonresponders in the high- and low-TLS/ICD score groups in patients from (B, E) PRJEB23709, (H, K) GSE100797, and (N, Q) GSE135222. Differences in TLS/ICD scores between responders and nonresponders in patients from (C, F) PRJEB23709, (I, L) GSE100797, and (O, R) GSE135222.

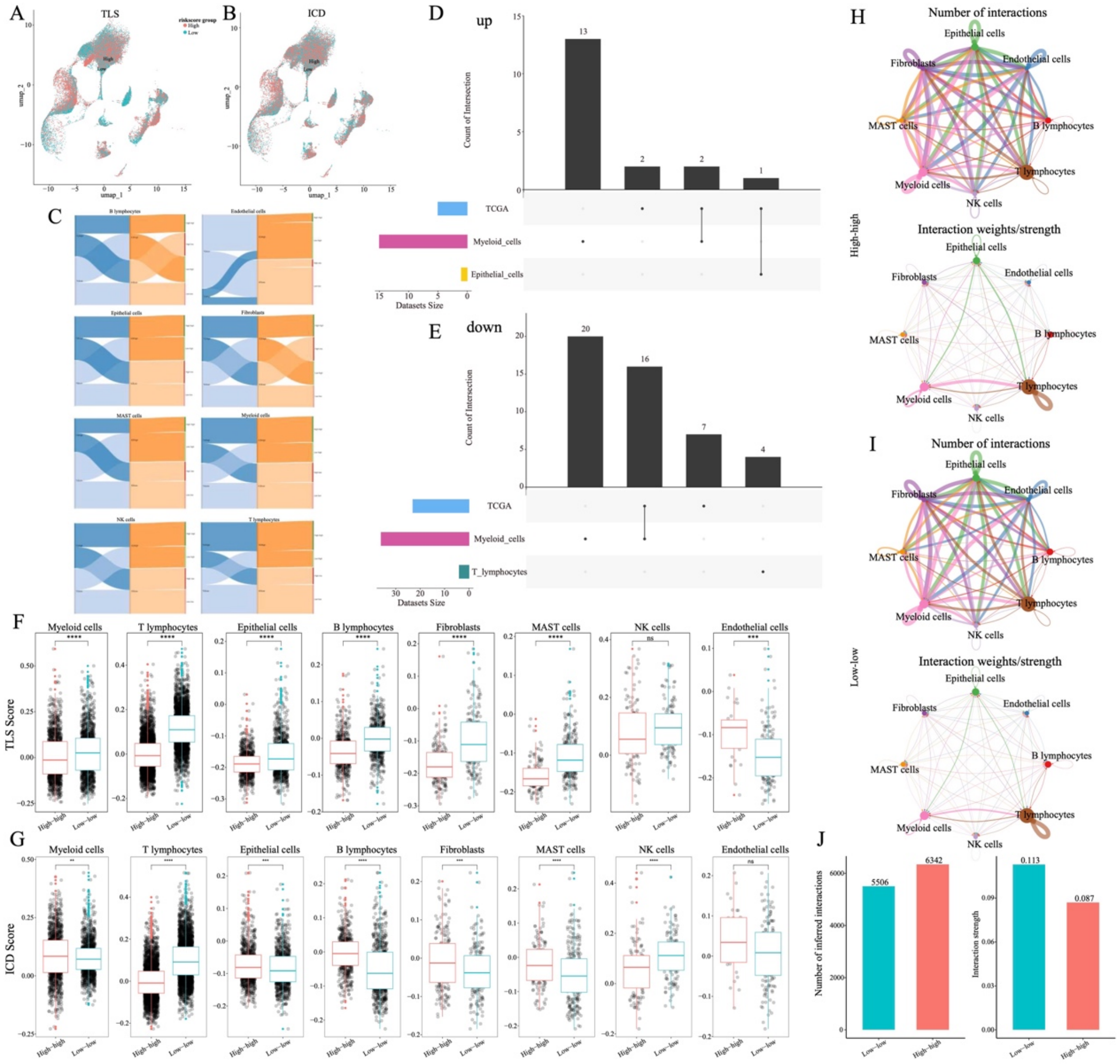

Supplementary Figure 10. Single-cell analysis of TLS- and ICD-related genes in LUAD. (A) UMAP plot showing TLS high- and low-risk-score cells. (B) UMAP plot showing ICD high- and low-risk-score cells. (C) Integration of TLS and ICD risk states to classify cells into high TLS/high ICD (HH), high TLS/low ICD (HL), low TLS/high ICD (LH), and low TLS/low ICD (LL) subgroups within each cell type. UpSet plots showing the intersections of (D) upregulated and (E) downregulated TLS- and ICD-related genes across cell types and the TCGA dataset. (F) Comparison of TLS scores and (G) ICD scores between HH and LL cell groups across cell types. Numbers and strengths of cell-cell interactions in the (H–I) HH and (J–K) LL groups, respectively. Differential interaction number and interaction strength between HH and LL groups are also shown.

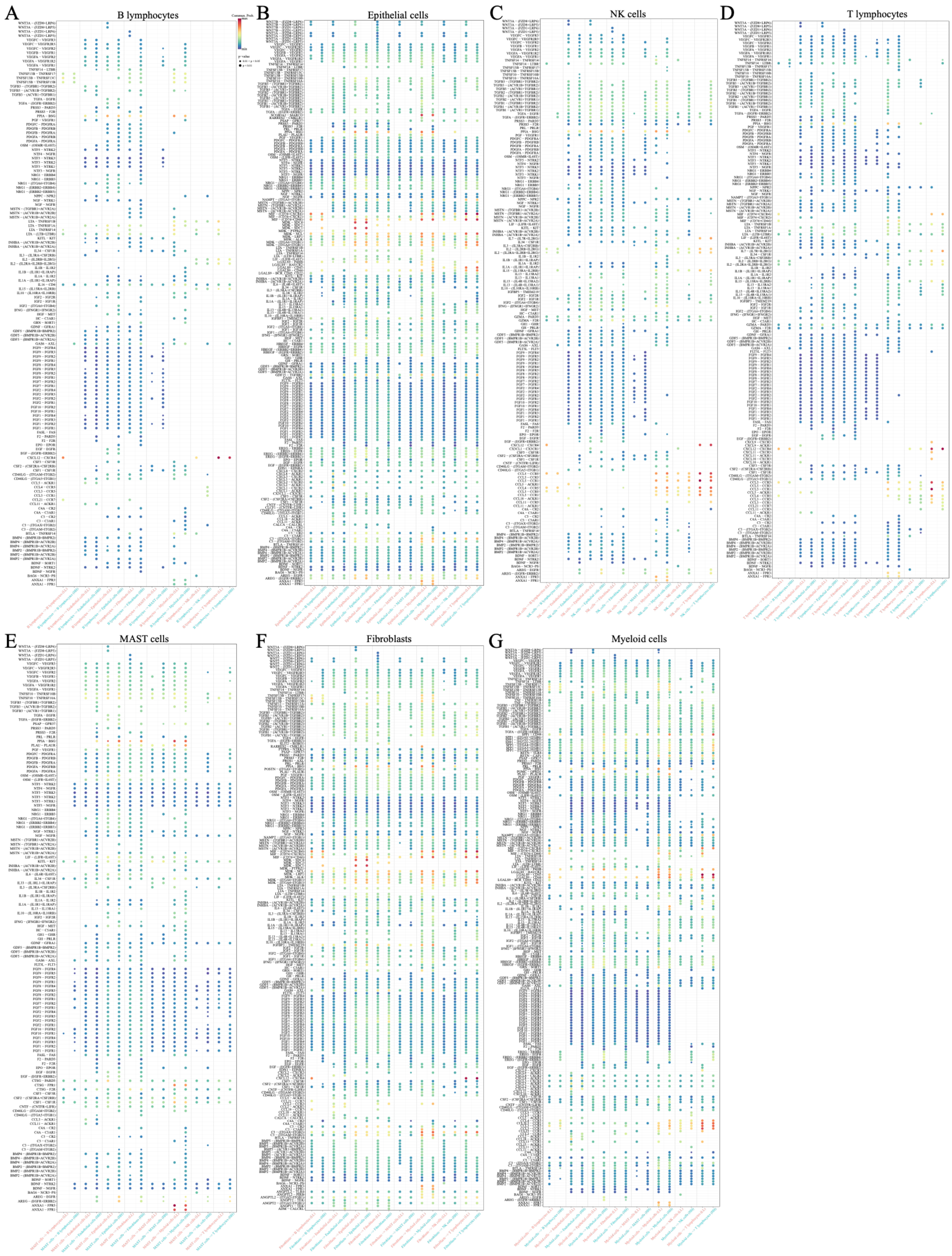

Supplementary Figure 11. Comparison of specific ligand–receptor interactions involving (A) B lymphocytes, (B) epithelial cells, (C) NK cells, (D) T lymphocytes, (E) mast cells, (F) fibroblasts, and (G) myeloid cells with other cell populations between the HH and LL groups.

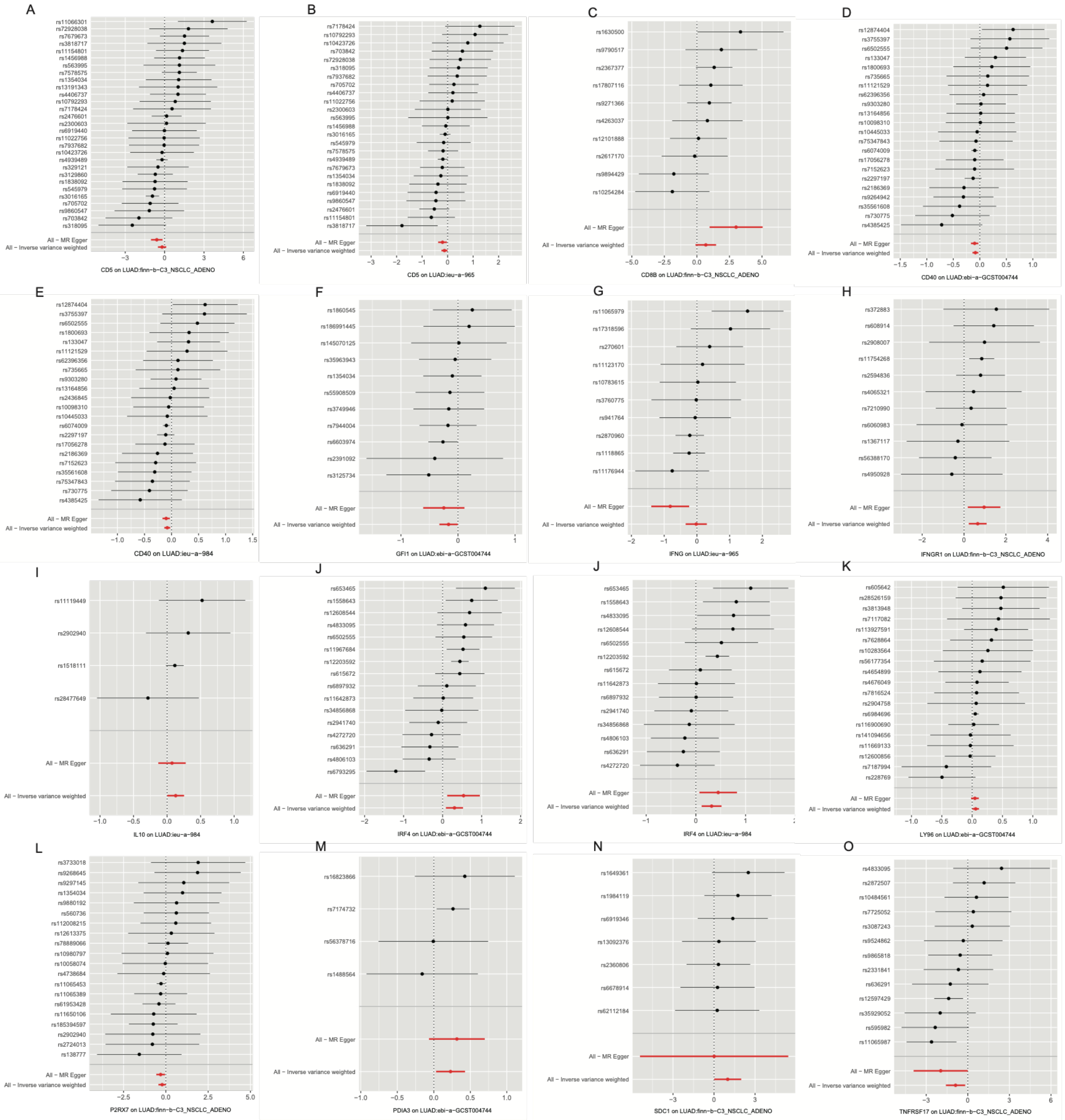

Supplementary Figure 12. Forest plots showing the causal associations between candidate genes and LUAD risk across four GWAS datasets.

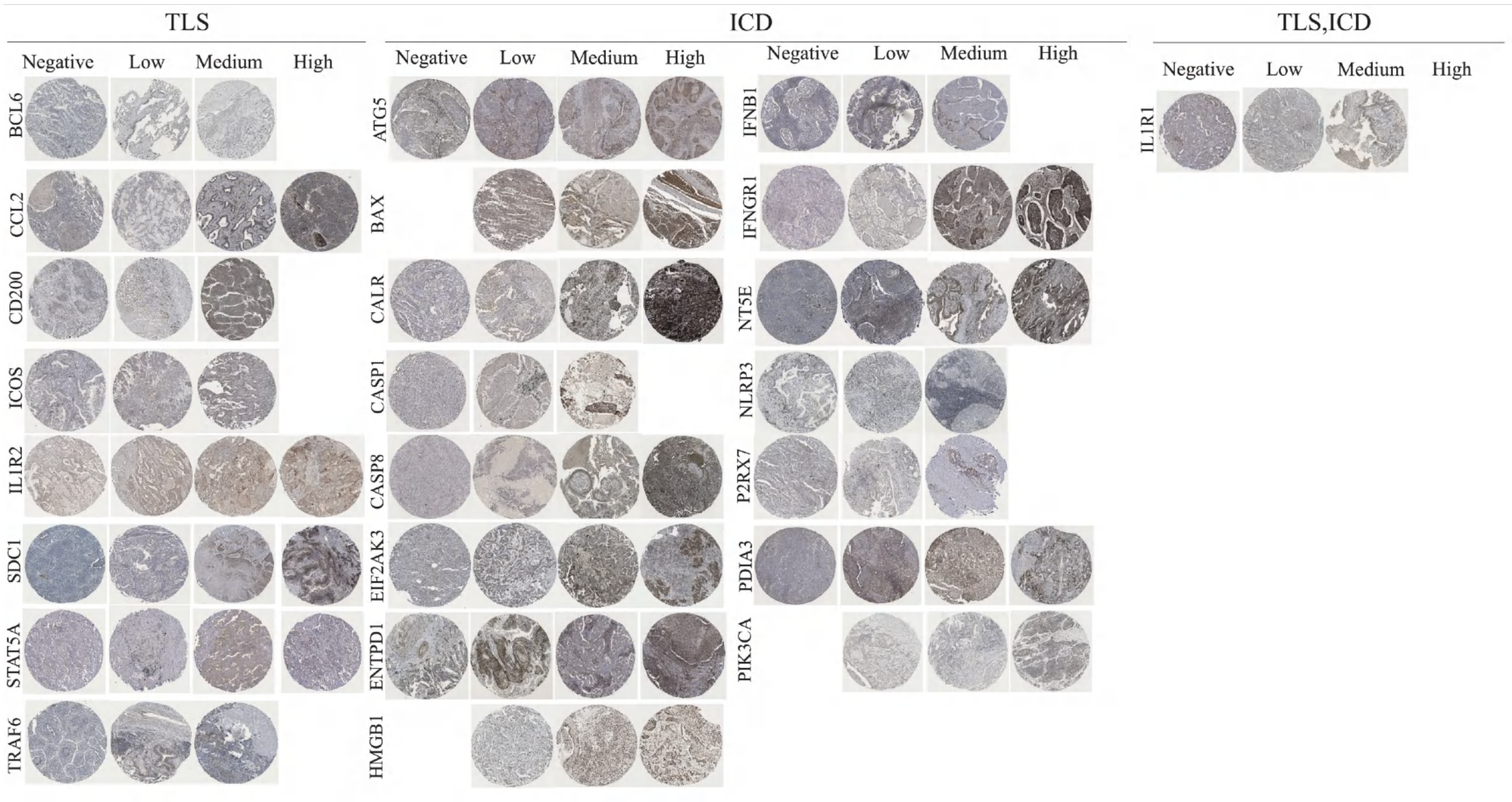

Supplementary Figure 13. Protein expression levels of TLS- and ICD-related genes in LUAD tumor tissues.

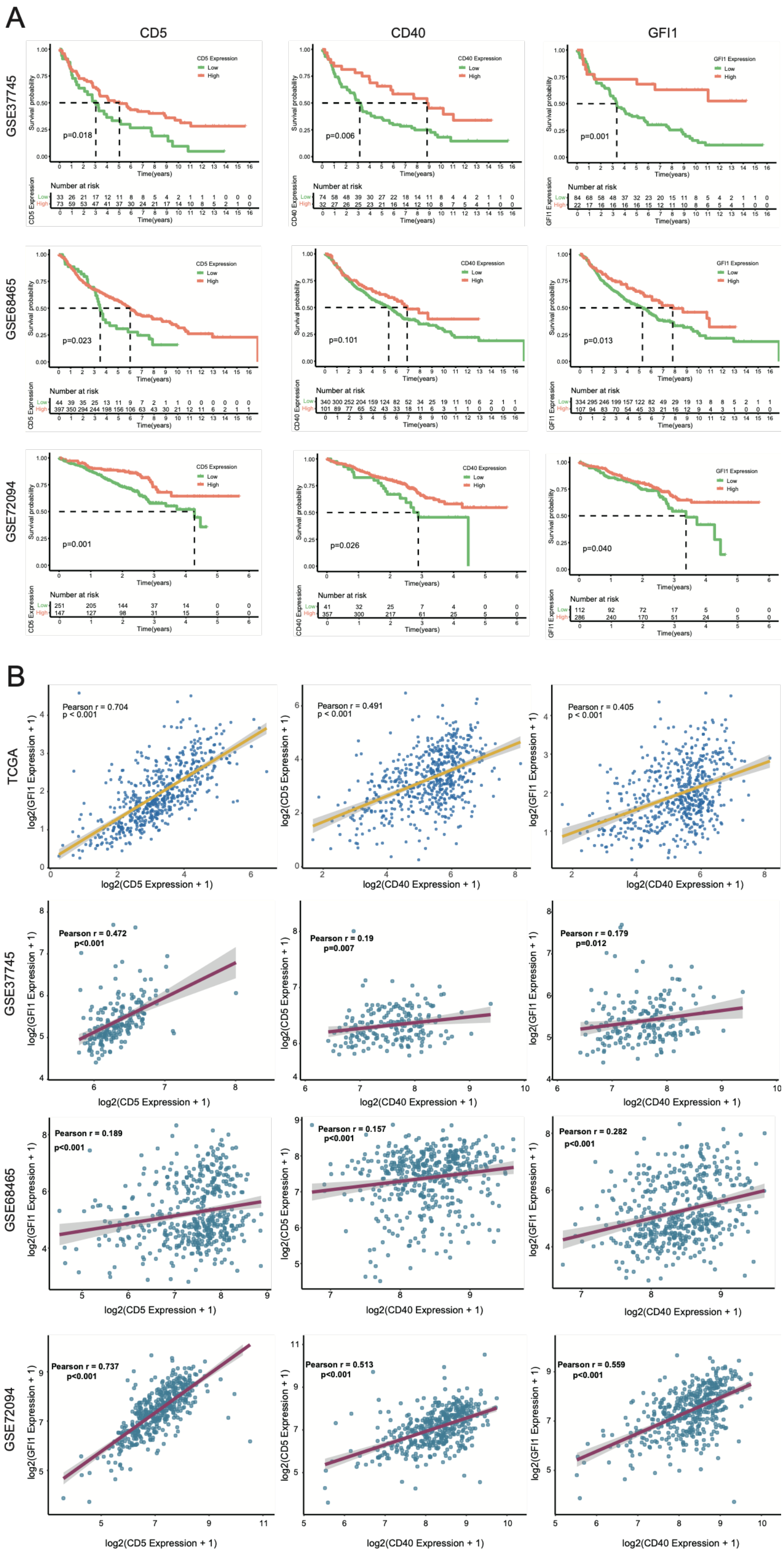

Supplementary Figure 14. (A) Kaplan–Meier survival curves showing the associations of CD40, CD5, and GFI1 with OS in the GSE37745, GSE68465, and GSE72094 cohorts. (B) Correlations among CD40, CD5, and GFI1 in the TCGA, GSE37745, GSE68465, and GSE72094 datasets.

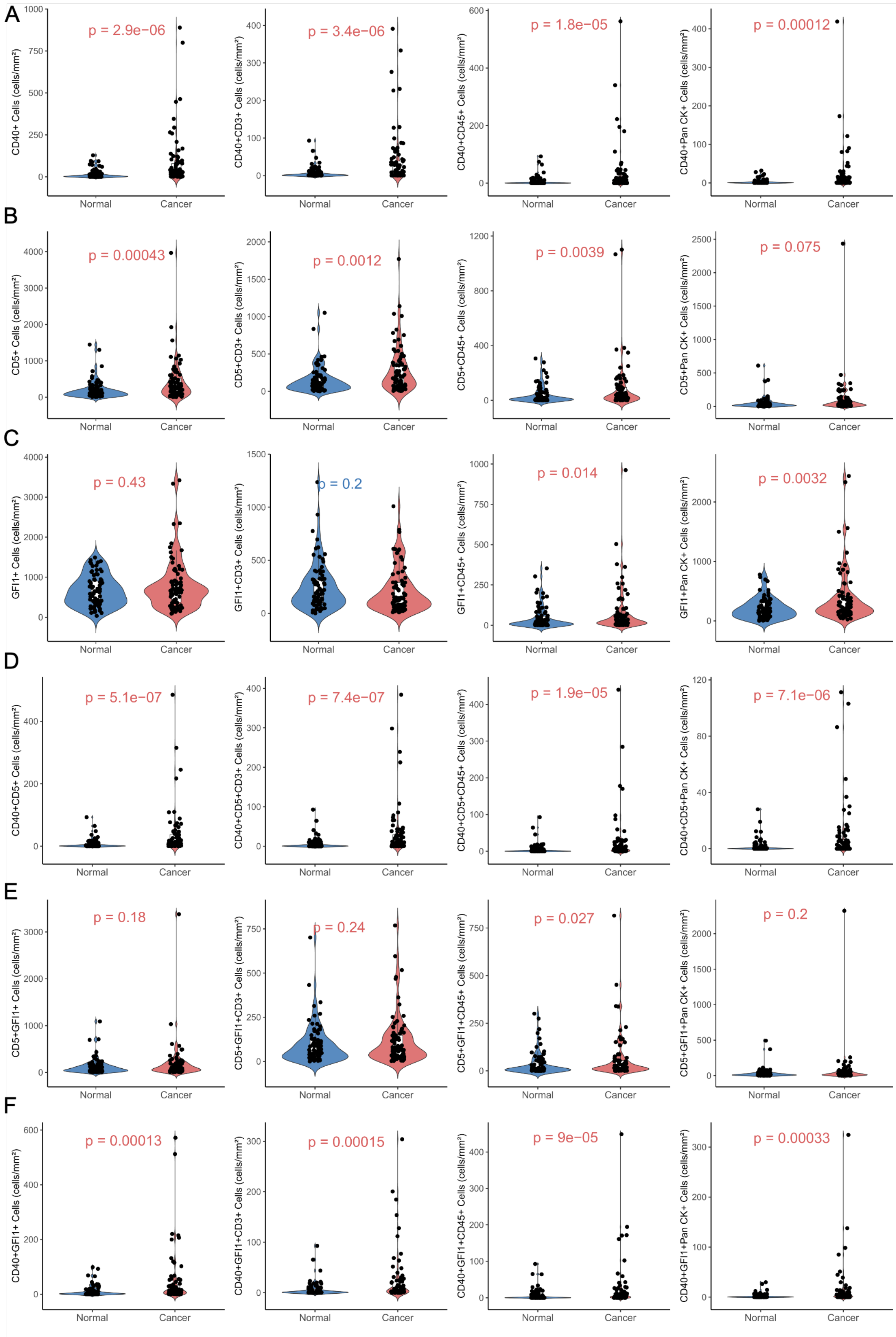

Supplementary Figure 15. Differential mIHC phenotypes of CD5, CD40, and GFI1 between paired normal and LUAD tissues. TSA-based multiplex immunofluorescence/multiplex immunohistochemistry (mIHC) was used to quantify

cell densities (cells/mm^2) of single-marker and composite phenotypes in paired normal and LUAD tissues. (A) CD40-related phenotypes, including CD40+, CD40+CD3+, CD40+CD45+, and CD40+PanCK+ cells. (B) CD5-related phenotypes, including CD5+, CD5+CD3+, CD5+CD45+, and CD5+PanCK+ cells. (C) GFI1-related phenotypes, including GFI1+, GFI1+CD3+, GFI1+CD45+, and GFI1+PanCK+ cells. (D) CD40–CD5 composite phenotypes, including CD40+CD5+, CD40+CD5+CD3+, CD40+CD5+CD45+, and CD40+CD5+PanCK+ cells. (E) CD5–GFI1 composite phenotypes, including CD5+GFI1+, CD5+GFI1+CD3+, CD5+GFI1+CD45+, and CD5+GFI1+PanCK+ cells. (F) CD40–GFI1 composite phenotypes, including CD40+GFI1+, CD40+GFI1+CD3+, CD40+GFI1+CD45+, and CD40+GFI1+PanCK+ cells. Red indicates tumor tissues and blue indicates normal tissues. *P* values indicate statistical significance for comparisons between paired normal and LUAD tissues.

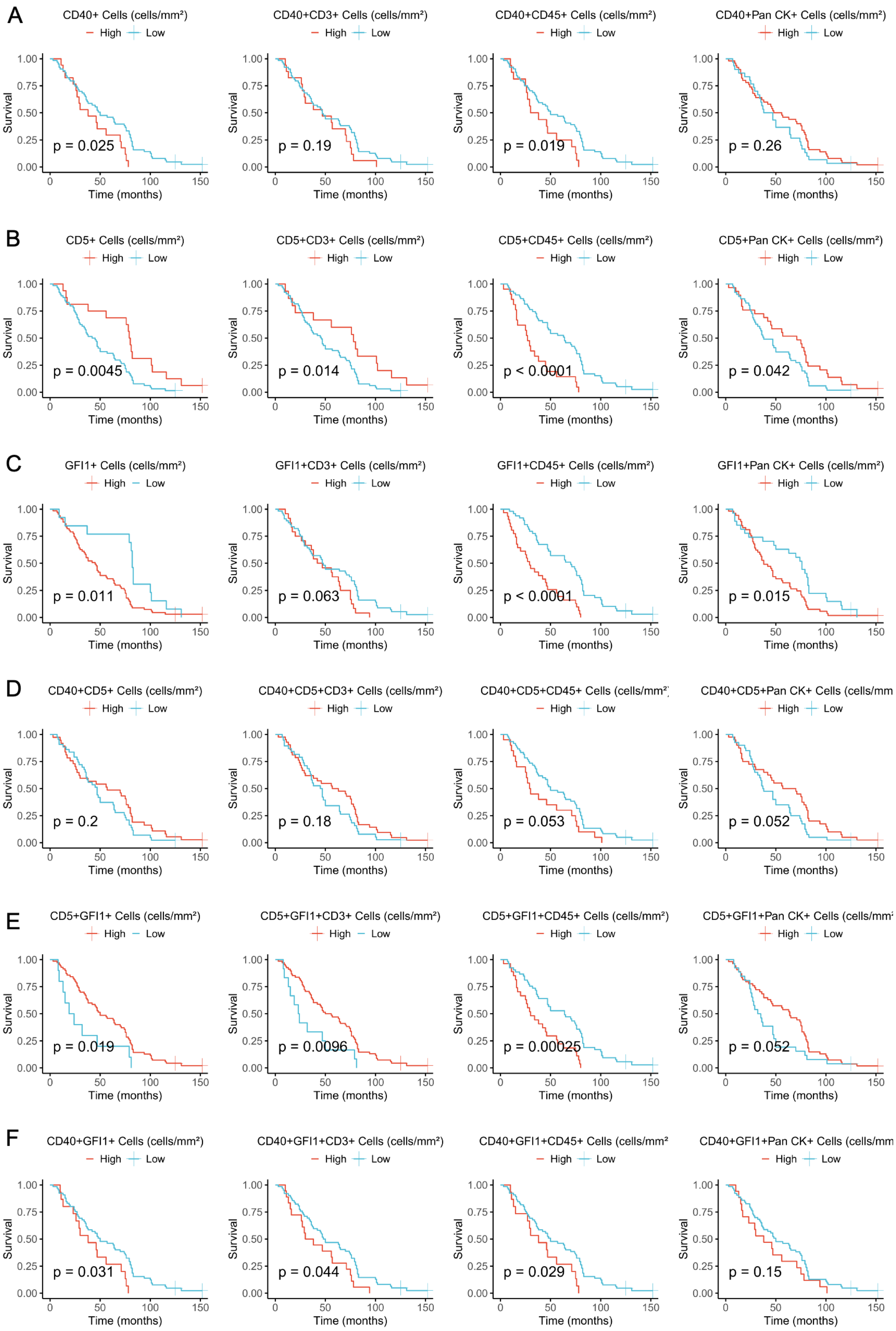

Supplementary Figure 16. Kaplan–Meier survival analyses of absolute mIHC phenotypes of CD5, CD40, and GFI1 in LUAD tissues. Kaplan–Meier curves show the associations between OS and the absolute densities (cells/mm²) of single-marker and

composite phenotypes quantified by TSA-based multiplex immunofluorescence/multiplex IHC in LUAD tissues. Patients were stratified into high- and low-expression groups according to the optimal cutoff value for each phenotype. (A) CD40-related phenotypes, including CD40+, CD40+CD3+, CD40+CD45+, and CD40+PanCK+ cells. (B) CD5-related phenotypes, including CD5+, CD5+CD3+, CD5+CD45+, and CD5+PanCK+ cells. (C) GFI1-related phenotypes, including GFI1+, GFI1+CD3+, GFI1+CD45+, and GFI1+PanCK+ cells. (D) CD40–CD5 composite phenotypes, including CD40+CD5+, CD40+CD5+CD3+, CD40+CD5+CD45+, and CD40+CD5+PanCK+ cells. (E) CD5–GFI1 composite phenotypes, including CD5+GFI1+, CD5+GFI1+CD3+, CD5+GFI1+CD45+, and CD5+GFI1+PanCK+ cells. (F) CD40–GFI1 composite phenotypes, including CD40+GFI1+, CD40+GFI1+CD3+, CD40+GFI1+CD45+, and CD40+GFI1+PanCK+ cells. Red lines indicate the high-expression group and blue lines indicate the low-expression group. *P* values were calculated using the log-rank test.

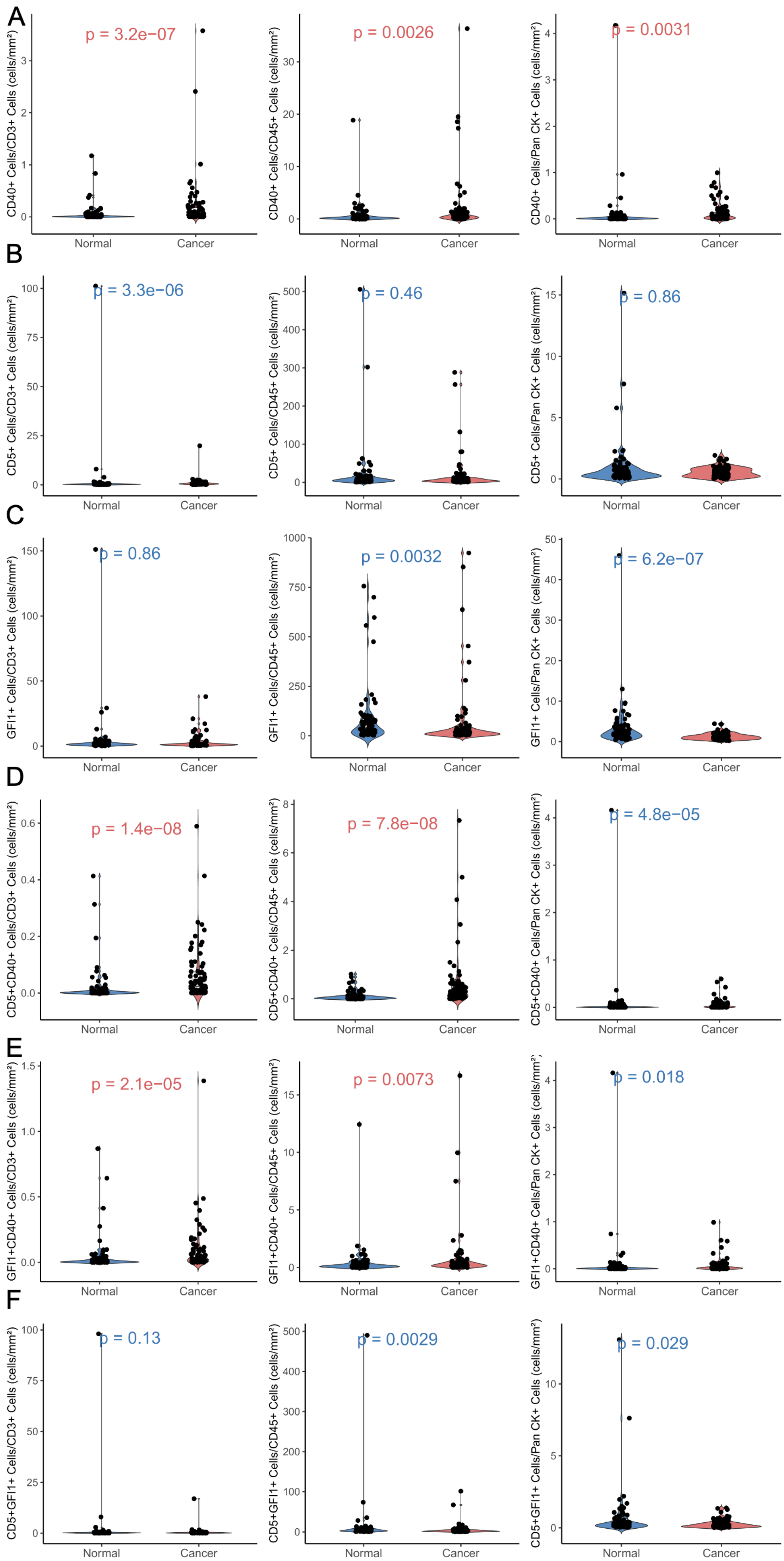

A
p = 3.2e−07
CD40+ Cells/CD3+ Cells (cells/mm²)
p = 0.0026
CD40+ Cells/CD45+ Cells (cells/mm²)
p = 0.0031
CD40+ Cells/Pan CK+ Cells (cells/mm²)
B
p = 3.3e−06
CD5+ Cells/CD3+ Cells (cells/mm²)
p = 0.46
CD5+ Cells/CD45+ Cells (cells/mm²)
p = 0.86
CD5+ Cells/Pan CK+ Cells (cells/mm²)
C
p = 0.86
GFI1+ Cells/CD3+ Cells (cells/mm²)
p = 0.0032
GFI1+ Cells/CD45+ Cells (cells/mm²)
p = 6.2e−07
GFI1+ Cells/Pan CK+ Cells (cells/mm²)
D
p = 1.4e−08
CD5+CD40+ Cells/CD3+ Cells (cells/mm²)
p = 7.8e−08
CD5+CD40+ Cells/CD45+ Cells (cells/mm²)
p = 4.8e−05
CD5+CD40+ Cells/Pan CK+ Cells (cells/mm²)
E
p = 2.1e−05
GFI1+CD40+ Cells/CD3+ Cells (cells/mm²)
p = 0.0073
GFI1+CD40+ Cells/CD45+ Cells (cells/mm²)
p = 0.018
GFI1+CD40+ Cells/Pan CK+ Cells (cells/mm²)
F
p = 0.13
CD5+GFI1+ Cells/CD3+ Cells (cells/mm²)
p = 0.0029
CD5+GFI1+ Cells/CD45+ Cells (cells/mm²)
p = 0.029
CD5+GFI1+ Cells/Pan CK+ Cells (cells/mm²)
Normal
Cancer

Supplementary Figure 17. Differential relative expression/compositional ratios of CD5, CD40, and GFI1 between paired normal and LUAD tissues. TSA-based multiplex immunofluorescence/multiplex IHC was used to quantify relative expression/compositional ratios of single-marker and composite phenotypes in paired normal and LUAD tissues. Ratios were calculated relative to CD3+, CD45+, or PanCK+ reference compartments. (A) CD40-related ratios, including CD40+/CD3+, CD40+/CD45+, and CD40+/PanCK+. (B) CD5-related ratios, including CD5+/CD3+, CD5+/CD45+, and CD5+/PanCK+. (C) GFI1-related ratios, including GFI1+/CD3+, GFI1+/CD45+, and GFI1+/PanCK+. (D) CD40–CD5 composite ratios, including CD40+CD5+/CD3+, CD40+CD5+/CD45+, and CD40+CD5+/PanCK+. (E) CD40–GFI1 composite ratios, including CD40+GFI1+/CD3+, CD40+GFI1+/CD45+, and CD40+GFI1+/PanCK+. (F) CD5–GFI1 composite ratios, including CD5+GFI1+/CD3+, CD5+GFI1+/CD45+, and CD5+GFI1+/PanCK+. Red indicates tumor tissues and blue indicates normal tissues. *P* values indicate statistical significance for comparisons between paired normal and LUAD tissues.

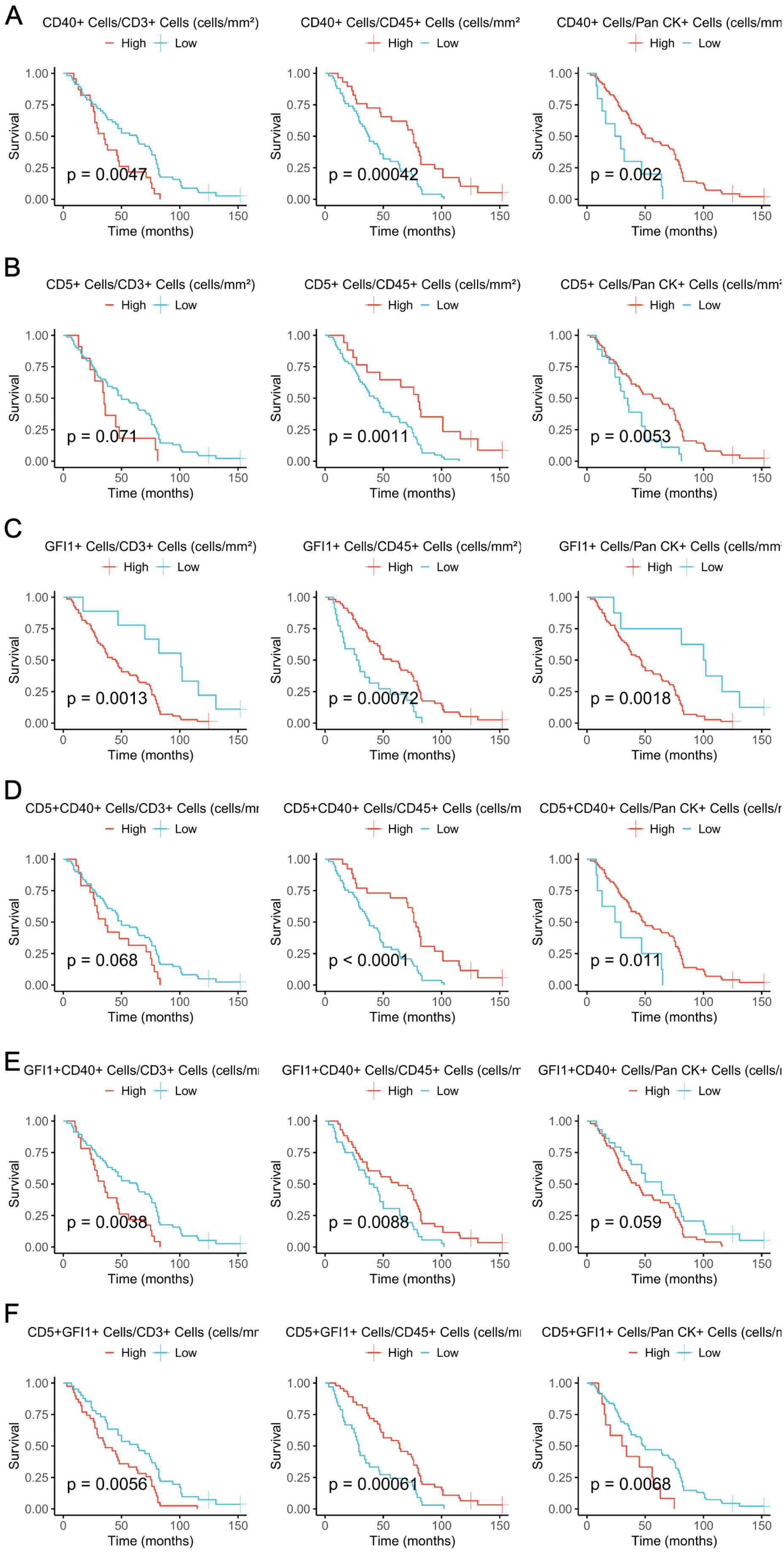
A
CD40+ Cells/CD3+ Cells (cells/mm²)
High Low
p = 0.0047
CD40+ Cells/CD45+ Cells (cells/mm²
High Low
p = 0.00042
CD40+ Cells/Pan CK+ Cells (cells/mm
High Low
p = 0.002
B
CD5+ Cells/CD3+ Cells (cells/mm²)
High Low
p = 0.071
CD5+ Cells/CD45+ Cells (cells/mm²)
High Low
p = 0.0011
CD5+ Cells/Pan CK+ Cells (cells/mm
High Low
p = 0.0053
C
GFI1+ Cells/CD3+ Cells (cells/mm²)
High Low
p = 0.0013
GFI1+ Cells/CD45+ Cells (cells/mm²)
High Low
p = 0.00072
GFI1+ Cells/Pan CK+ Cells (cells/mm
High Low
p = 0.0018
D
CD5+CD40+ Cells/CD3+ Cells (cells/mı
High Low
p = 0.068
CD5+CD40+ Cells/CD45+ Cells (cells/m
High Low
p < 0.0001
CD5+CD40+ Cells/Pan CK+ Cells (cells/ı
High Low
p = 0.011
E
GFI1+CD40+ Cells/CD3+ Cells (cells/mı
High Low
p = 0.0038
GFI1+CD40+ Cells/CD45+ Cells (cells/m
High Low
p = 0.0088
GFI1+CD40+ Cells/Pan CK+ Cells (cells/ı
High Low
p = 0.059
F
CD5+GFI1+ Cells/CD3+ Cells (cells/mr
High Low
p = 0.0056
CD5+GFI1+ Cells/CD45+ Cells (cells/mı
High Low
p = 0.00061
CD5+GFI1+ Cells/Pan CK+ Cells (cells/n
High Low
p = 0.0068
Survival
1.00
0.75
0.50
0.25
0.00
0
50
100
150
Time (months)

Supplementary Figure 18. Kaplan–Meier survival analyses of relative expression/compositional ratios of CD5-, CD40-, and GFI1-related phenotypes in LUAD. Kaplan–Meier curves show the associations between OS and relative expression/compositional ratio metrics quantified by TSA-based multiplex immunofluorescence/multiplex IHC in LUAD tissues. Patients were stratified into high- and low-expression groups according to the optimal cutoff value for each ratio metric. Ratios were calculated relative to CD3+, CD45+, and PanCK+ reference compartments. (A) CD40-related ratios, including CD40+/CD3+, CD40+/CD45+, and CD40+/PanCK+. (B) CD5-related ratios, including CD5+/CD3+, CD5+/CD45+, and CD5+/PanCK+. (C) GFI1-related ratios, including GFI1+/CD3+, GFI1+/CD45+, and GFI1+/PanCK+. (D) CD40–CD5 composite ratios, including CD40+CD5+/CD3+, CD40+CD5+/CD45+, and CD40+CD5+/PanCK+. (E) CD40–GFI1 composite ratios, including CD40+GFI1+/CD3+, CD40+GFI1+/CD45+, and CD40+GFI1+/PanCK+. (F) CD5–GFI1 composite ratios, including CD5+GFI1+/CD3+, CD5+GFI1+/CD45+, and CD5+GFI1+/PanCK+. Red lines indicate the high-expression group and blue lines indicate the low-expression group. *P* values were calculated using the log-rank test.

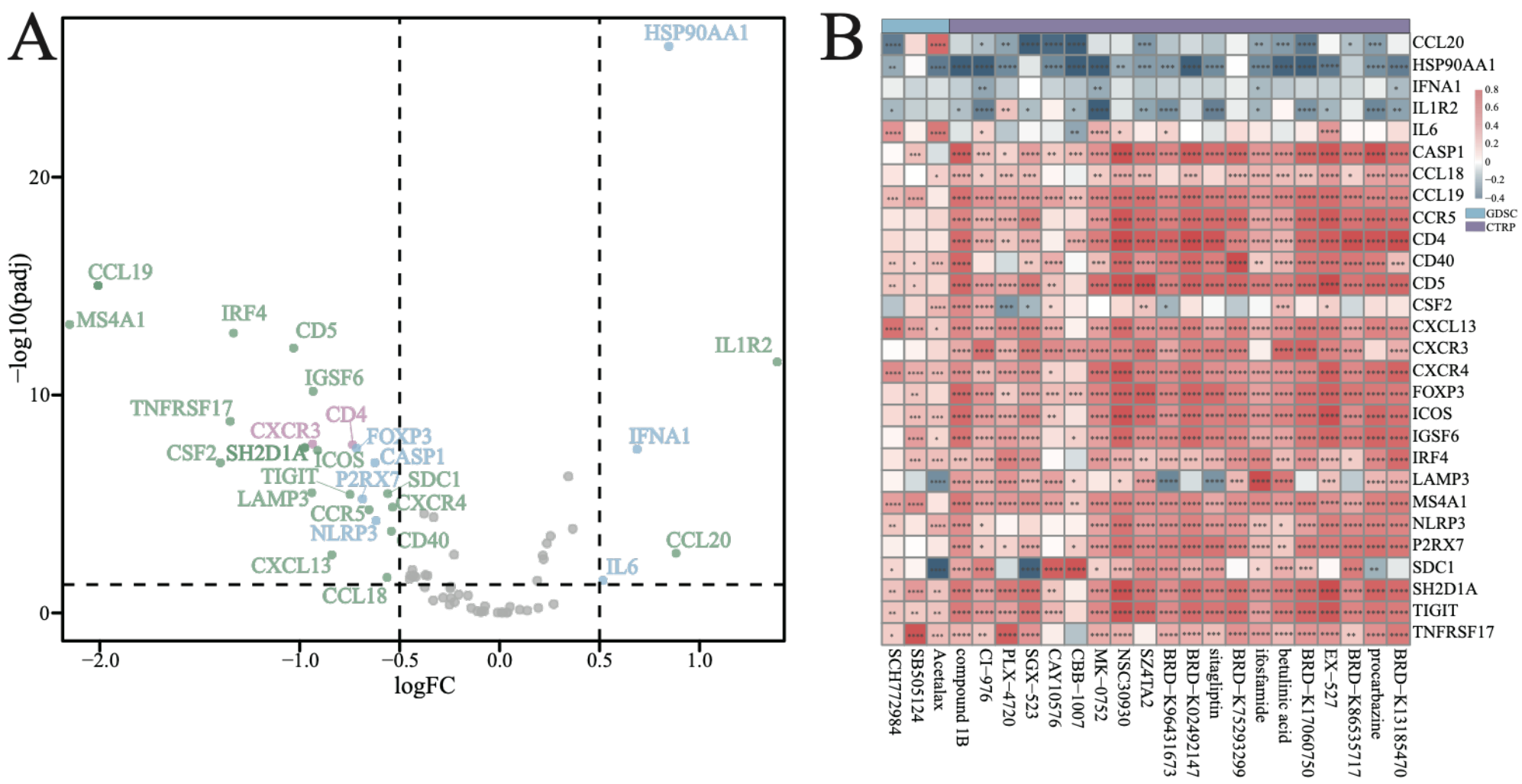

Supplementary Figure 19. Drug prediction analysis for the HH subgroup. (A) Differential expression of TLS- and ICD-related genes between the HH and LL groups. The volcano plot displays the significantly upregulated and downregulated genes in HH tumors relative to LL tumors. (B) Correlation heatmap between candidate compounds from the GDSC and CTRP databases and differentially expressed TLS- and ICD-related genes. The heatmap illustrates the potential associations between gene-expression patterns and predicted drug sensitivity, which were used to prioritize compounds with possible therapeutic relevance for the HH subgroup.

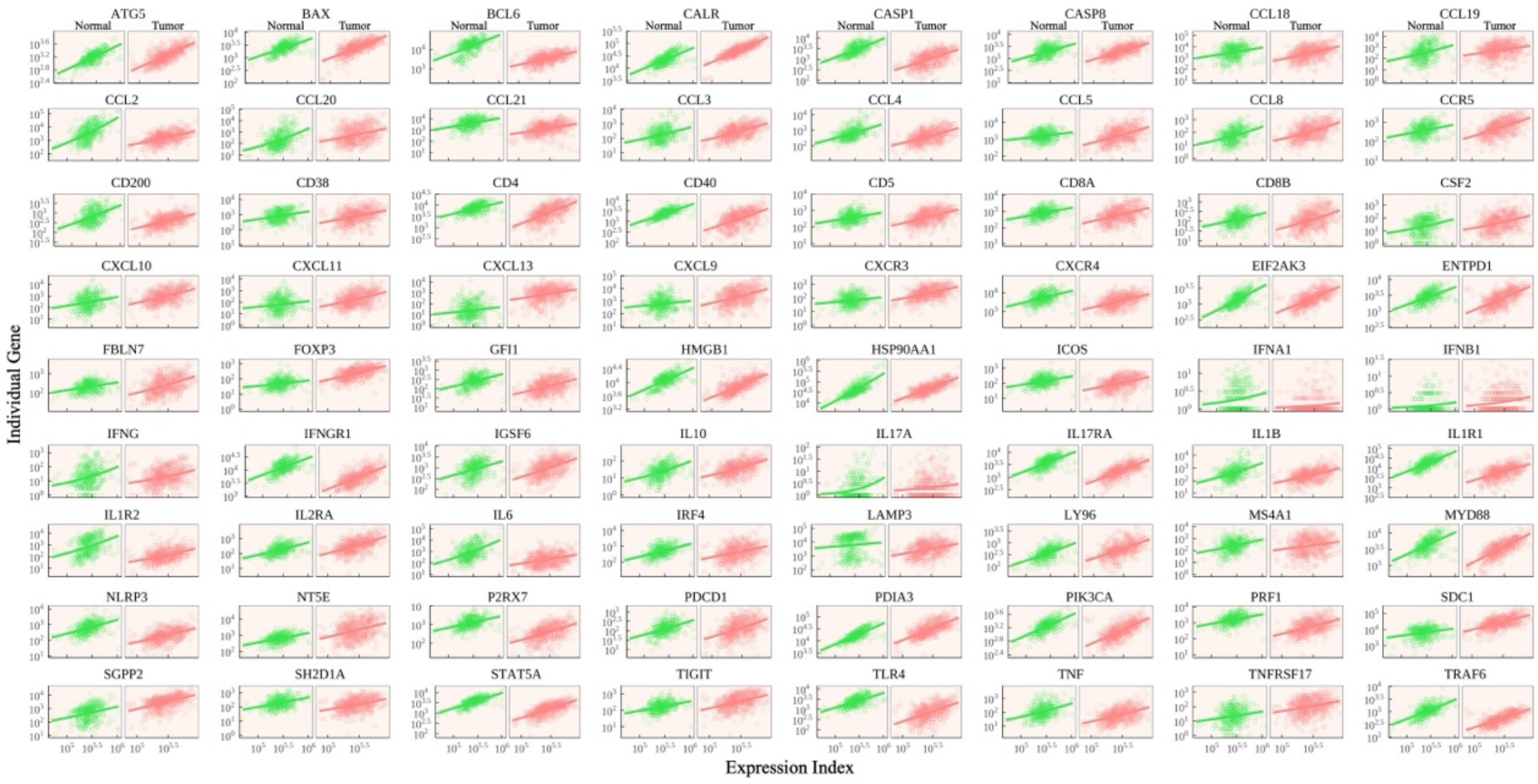

Supplementary Figure 20. Fitting curves of the power equation to gene expression values across normal patients (green) and tumor patients (red).

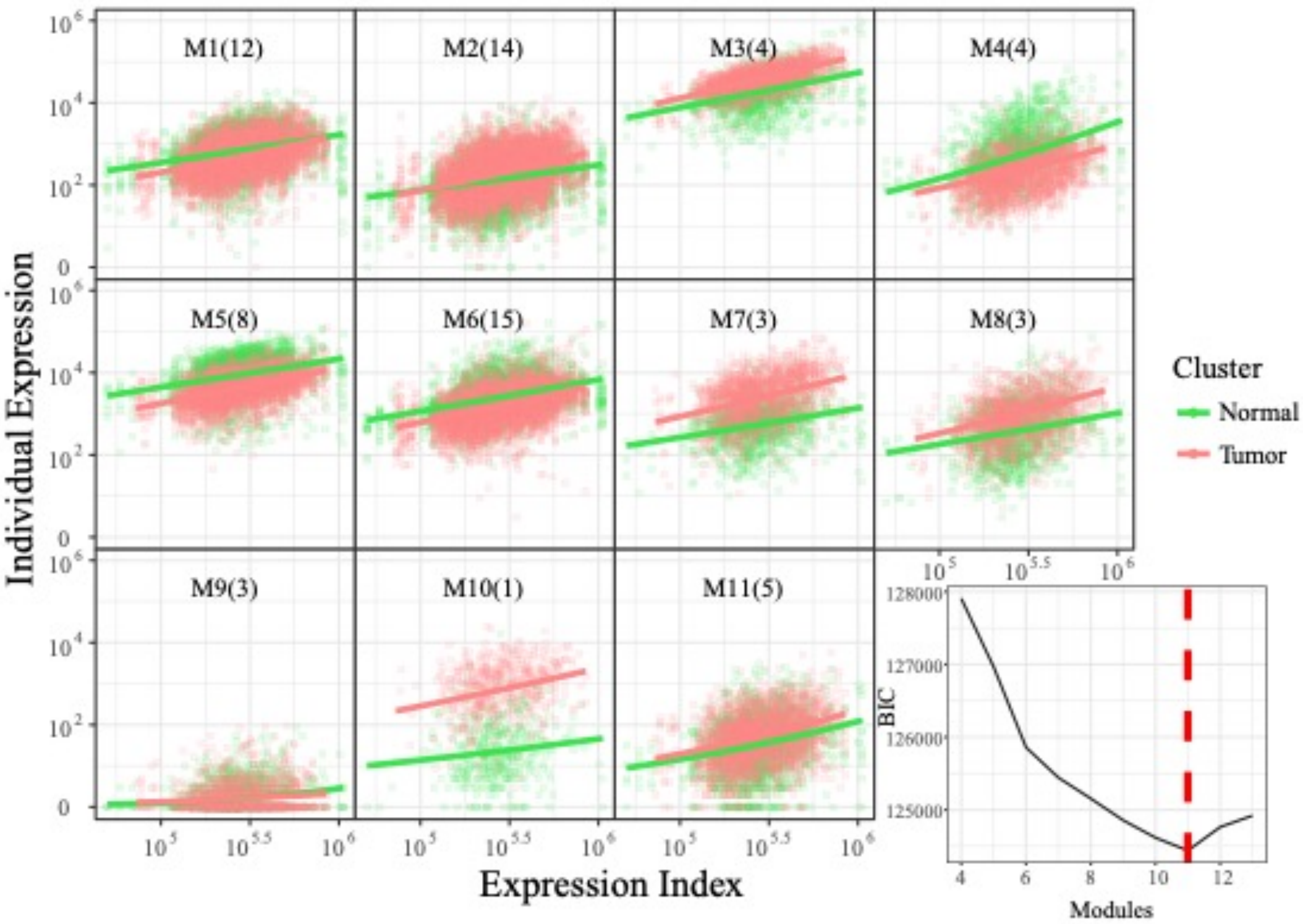

Supplementary Figure 21. Module classification of TLS- and ICD-related genes in normal patients (green) and tumor patients (red). The optimal number of clusters was determined according to the Bayesian information criterion (BIC).

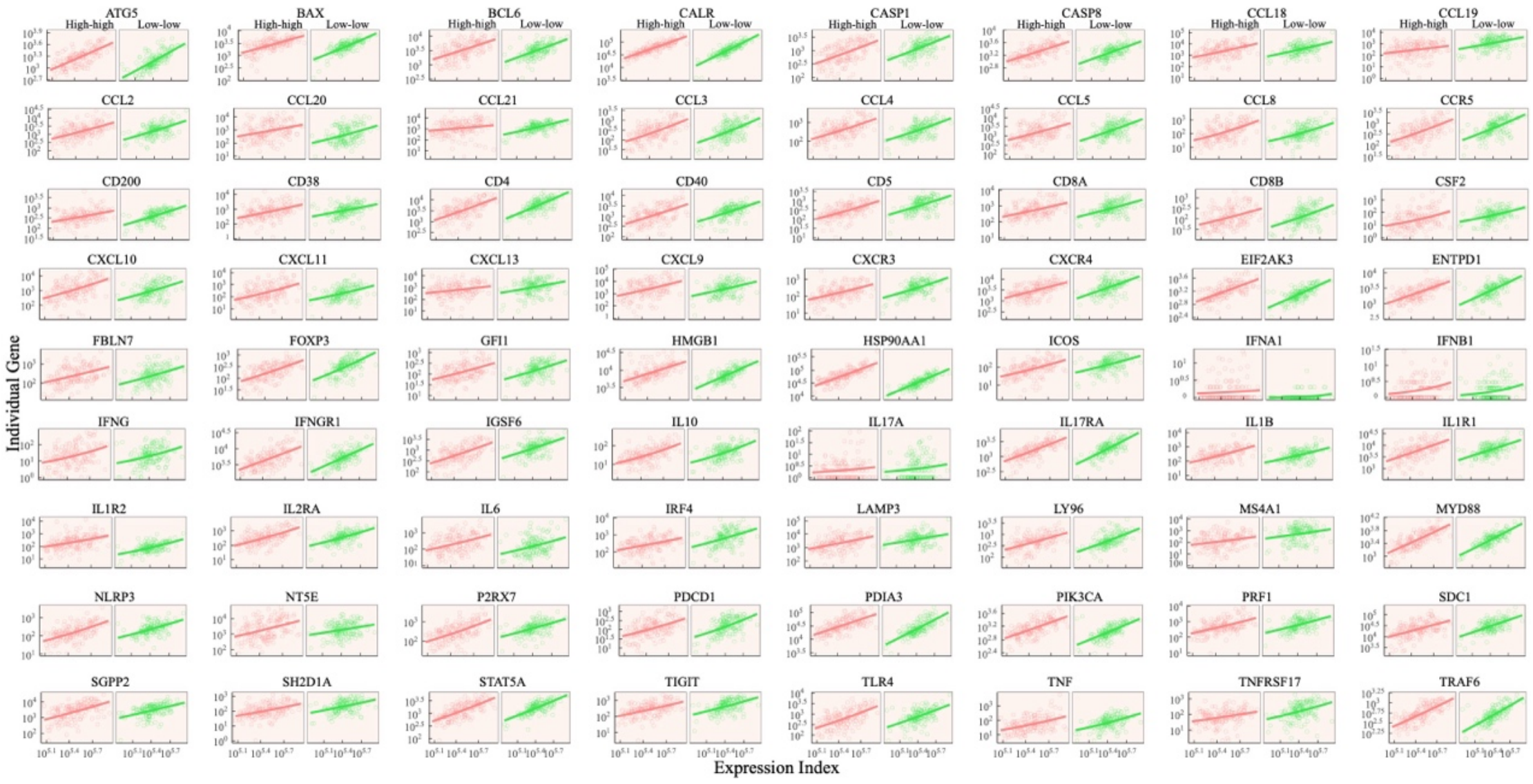

Supplementary Figure 22. Fitting curves of the power equation to gene expression values across HH patients (red) and LL patients (green).

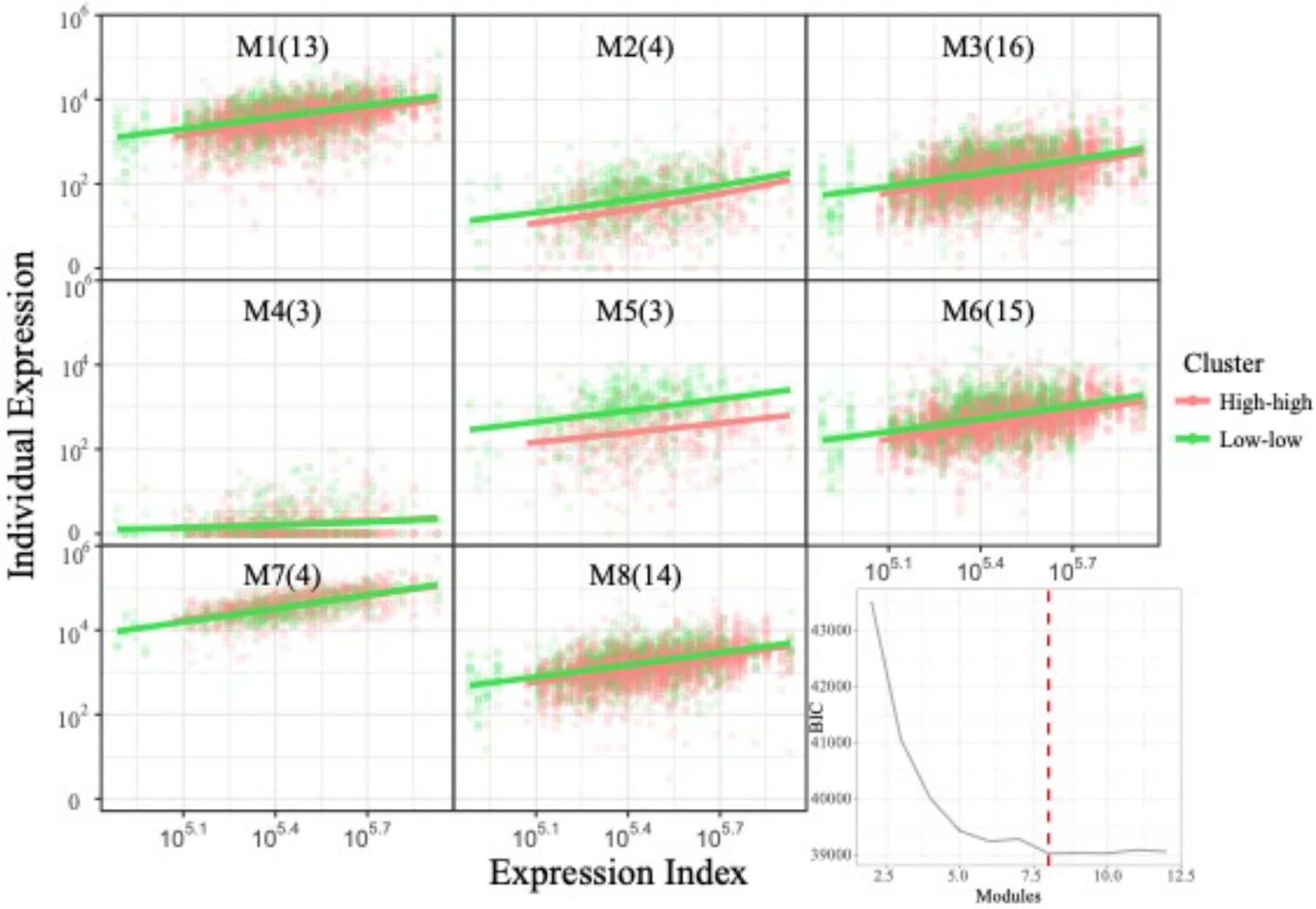

Supplementary Figure 23. Module classification of TLS- and ICD-related genes in LL patients (green) and HH patients (red). The optimal number of clusters was determined according to the BIC.
